# Visual prototypes in the ventral stream are attuned to complexity and gaze behavior

**DOI:** 10.1038/s41467-021-27027-8

**Published:** 2021-11-18

**Authors:** Olivia Rose, James Johnson, Binxu Wang, Carlos R. Ponce

**Affiliations:** 1 Department of Neuroscience, Washington University School of Medicine, St. Louis, MO USA; 2 Department of Neurobiology, Harvard Medical School, Boston, MA USA

**Keywords:** Neural decoding, Object vision

## Abstract

Early theories of efficient coding suggested the visual system could compress the world by learning to represent features where information was concentrated, such as contours. This view was validated by the discovery that neurons in posterior visual cortex respond to edges and curvature. Still, it remains unclear what other information-rich features are encoded by neurons in more anterior cortical regions (e.g., inferotemporal cortex). Here, we use a generative deep neural network to synthesize images guided by neuronal responses from across the visuocortical hierarchy, using floating microelectrode arrays in areas V1, V4 and inferotemporal cortex of two macaque monkeys. We hypothesize these images (“prototypes”) represent such predicted information-rich features. Prototypes vary across areas, show moderate complexity, and resemble salient visual attributes and semantic content of natural images, as indicated by the animals’ gaze behavior. This suggests the code for object recognition represents compressed features of behavioral relevance, an underexplored aspect of efficient coding.

## Abstract (web summary)

Visual recognition depends on the ability to extract specific shape and colour features from complicated natural scenes. Here, the authors show that neurons along the object-recognition cortical pathway encode information-concentrating features of moderate complexity and of behavioural relevance.

## Introduction

The brain faces multiple constraints when representing the visual world, from metabolic costs^1^, wiring constraints^2^, and the need to separate signal from noise in retinal inputs. There are different hypotheses for optimizing visual coding under these constraints. For example, early frameworks noted that since pixel-by-pixel representations of visual scenes are redundant, neurons should learn to represent features that are information-rich, such as contours^3^. Subsequently, it was found that neurons in early visual cortex encode information about contours and corners^4^, with neurons representing Gabor-like filters of different orientations and spatial frequencies^5^ (a finding with strong theoretical support in computational simulations of efficient coding^6,7^). Beyond simple contours, there remains an incomplete understanding of other visual motifs used by neurons for object recognition. One hypothesis is that the visual system must organize sensory information to build up an “internal model of the environment,” centered around diagnostic motifs of visual objects of “particular key significance for the animal,” as postulated by Horace Barlow^8^. Further, these motifs should not only be related to external features of the visual environment, but also to the animal’s actions within it^9^— for example, related to behaviors such as saccadic eye movements that bring the fovea to salient regions of a scene^10^. If these internal model motifs exist, they must be simpler than visual scenes themselves—the critical question being, how much simpler?

Here, we set out to extract direct samples of the neural code for object representation, and measured properties related to their information content. Electrophysiological responses from cortical sites at the border of V1 and V2, in V4, and in inferotemporal cortex (IT) were used for neuron-guided image synthesis. Specifically, using neuronal responses, an evolutionary algorithm^11^ optimized inputs to an image-generating deep neural network, a network that employed up-convolution operations to synthesize images from a latent variable vector^12^ (the “input space”). These synthetic images contain visual features that evoked the highest observed neuronal activity at that site. We use independently trained neural networks to interpret images semantically, finding that V1/V2 sites encode more monochrome contours than V4 and IT, while IT sites encode more visual attributes related to animals than V1/V2 and V4 (but less reliably for other semantic categories such as *food*). We measure the compressibility of these activating images, as well as the relative number of parts needed to characterize them, finding they are intermediate in complexity compared to standard artificial stimulus sets and AlexNet representations. Finally, we look for a link between these synthetic images and the animals’ spontaneous behavior, discovering that these highly activating images are related to regions that also draw the animals’ gaze. We conclude that ventral stream neurons encode information-concentrating features present in the natural visual world, features marked by their relevance to the organism.

## Results

### Characterization of response selectivity

**Fig. 1:**
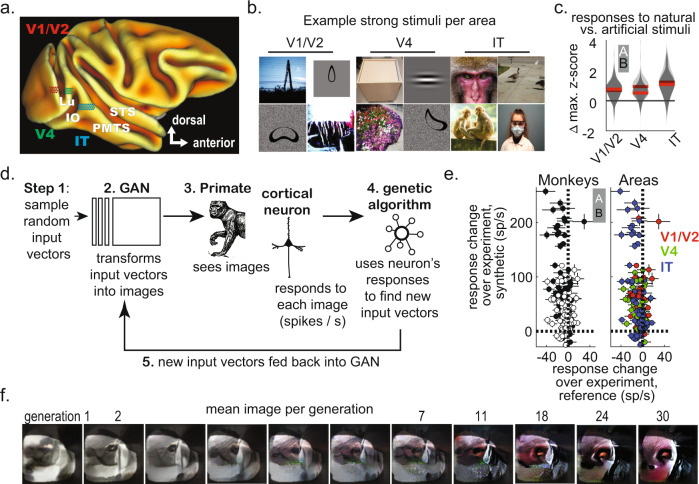
Characteristics of the neuronal populations. **a** Sketch illustrating the location of floating microelectrode arrays relative to different occipito-temporal sulci (Lu: lunate, IO: inferior occipital, PMTS: posterior middle temporal, and STS: superior temporal), and their functional designation (V1/V2: red; V4: green; IT: blue). **b** Examples of images that elicited high responses from V1/V2, V4, and IT sites (some photographs are comparable replacements to the original stimuli, changed due to copyright issues, see our GitHub repository for originals^72^). **c** Response difference between natural photographs and artificial images for sites in different cortical areas per monkey (A, white; B, black, red line shows median). Neuronal spike rate responses were z-scored across images but within each site, then differences (∆) measured from the z-scored responses shown as violin plots (red line = median values, *n* = array sites tested across days; monkey A, V1/V2, 150; V4, 67; PIT, 93; monkey B, V1/V2, 34; V4, 15; IT, 33). Each ∆ value was obtained by subtracting the maximum z-score to artificial images from the maximum z-score to natural images. **d** Experimental workflow for image synthesis, showing one full cycle of image generation. **e** Difference in mean neuronal response from the beginning to the end of the experimental session (events per second; mean ± SE); the y-axis shows the difference in mean response to the synthetic images over generations, and the x-axis shows the difference in mean response to the reference images over generations (because the reference images were not changing, the neurons showed adaptation during the experimental session). The left scatterplot shows results per monkey, and the right, for the cortical area (same colors as in panel a). *n* = experiments per animal, 127 in monkey A; 125 experiments in B. **f** One example image synthesis experiment using responses from a posterior IT site in Monkey B; each image corresponds to the average genotype of a given generation (black number on top). Mask shows the approximate location of the receptive field center. Source data are provided as a Source Data file. Depicted individuals have consented for publication, and have seen these photos in the context of the publication.

We recorded from 128 unique cortical sites in two male monkeys (*Macaca mulatta*) in regions conventionally described as the border of V1/V2^13^, V4^14,15^, and posterior/central IT^16^ (Fig. 1a, Methods). Most sites responded best to images presented peri-foveally (Supplementary Fig. 1, Table 1). To characterize the visual tuning of each site, we used a set of 754 unique stimuli consisting of natural and artificial images (ranging from photographs sampled from ImageNet^17^, Google image search, and other sources, to artificial images such as straight and curved Gabor patches, Fig. 1b). We found that most visually responsive sites were modulated by image identity (89.2 ± 5.1% and 93.5 ± 4.4% of V1/V2 sites in monkeys A and B showed image identity selectivity, under a criterion of *P* < 0.05 with one-way ANOVA, after false discovery correction; this was also true for 73.7 ± 3.5% and 100.0 ± 0.0 of V4 sites in A and B, and 49.3 ± 3.1% and 64.0 ± 6.7 of IT sites in A and B).

**Table 1:** Estimates of receptive field centers for both animals, per visual area.

	V1	V4	IT
Monkey A			
Distribution of RF centers in visual degrees (°), reported as (horizontal, vertical) at 25th, 50th, and 75th %iles	(−0.1, −0.2)	(−0.9, −2.3)	(−1.2, −3.0)
	(−0.0, −0.1)	(−0.7, −1.7)	(−1.0, −2.7)
	(0.0, −0.1)	(−0.3, −1.3)	(−0.3, −1.9)
Eccentricity (mean ± SEM)	0.27 ± 0.09	1.83 ± 0.10	2.93 ± 0.21
Gaussian width (pooled across horizontal and vertical values)	1.39, 1.47, 2.56	2.00, 2.34, 5.24	2.84, 3.62, 3.99
Monkey B			
Distribution of RF centers in visual degrees	(−0.1, −0.4)	(−2.0, −2.6)	(−1.3, 0.3)
	(−0.0, −0.3)	(−0.8, −1.7)	(−1.0, 0.6)
	(0.1, −0.2)	(−0.2, −0.5)	(−0.8, 0.8)
Eccentricity (mean ± SEM)	0.63 ± 0.09	2.03 ± 0.09	1.56 ± 0.17
Gaussian width (pooled across horizontal and vertical values)	1.54, 2.18, 2.50	1.56, 2.54, 3.38	2.47, 2.80, 3.16

Sites in every area responded more strongly to photographs than to traditional artificial stimuli such as Gabor patches, curved contour objects, and spirals. We computed each site’s maximum response to all photographs and to all artificial line objects (responses were z-scores of spike rates, to allow comparisons across experiments), and then measured the difference between both maximum values. These differences were then parsed per visual area, and tested for statistical significance using a Kruskal-Wallis test (with areas as grouping variables) or Wilcoxon rank-sum tests (for comparing V1/V2 and IT differences, with the simple difference formula [i.e. fraction of all cross-sample pairwise differences that are positive minus the fraction that are negative] as the central effect size, see Kerby et al.^18^ for details). We found that in monkey A, the median difference in maximum z-score values (to photographs minus artificial) were as follows: in V1-V2, 0.70 ± 0.06, V4, 0.93 ± 0.09 and PIT, 1.25 ± 0.08 (probability of seeing a difference in medians this large or larger assuming the null hypothesis of equal medians is true was *P* = 1.6 × 10^−8^, Kruskal-Wallis test, *χ*^*2*^ = 35.9, deg. freedom = 307); for monkey B, the values were V1-V2: 0.80 ± 0.13, V4: 0.59 ± 0.16, PIT: 1.07 ± 0.10 (*P* = 1.7 × 10^−2^, *χ*^*2*^ = 8.2, df = 79). We also asked if there was a statistical difference between V1/V2 vs. IT sites using a two-sample test and found that the probability of a difference as large or larger under the null hypothesis of equal medians was *P* = 4.8 × 10^−9^ (monkey A, Wilcoxon rank-sum test, simple-difference = −0.45) and *P* = 0.04 (monkey B, simple-difference = −0.29, Fig. 1c, Methods).

Although Gabor patches were less effective than natural images in general, we confirmed that V1/V2 sites were better modulated by Gabor patches than other areas (the percentages of sites that were strongly modulated by Gabor patches, per one-way ANOVA, *P* < 0.05, after false discovery adjustment, were 75% and 81% for V1/V2 [monkeys A and B, *N* = 16 sites each, F-statistics ranging from 0.9–40.9], 31% and 69% for V4 (*N* = 16 sites each, F-statistics ranging from 0.6–9.5) and 6% and 22% for IT (*N* = 32 sites each, F-statistics ranging from 0.1–17.5).

The object-recognition system from Google Cloud Vision^16^ provided a set of labels for images independent of the image origin. The inferred labels were more descriptive and pulled from a wider repertoire than the ImageNet database used to train the generator^19^. We found IT sites showed strong responses to pictures with labels related to monkeys, humans, and other mammals; in contrast, V4 and V1/V2 sites were less specific, responding best to natural images across a broad set of categories including “water,” “plants,” and “national parks” (Table 2). Thus, IT selectivity was focused on animal features, while V4 and V1/V2 selectivity covered more generic features. To clarify the relationship of these generic features with V1/V2 orientation tuning, we used the histogram-of-gradients (HOG) algorithm to define an orientation dominance index for every image; we found that V1/V2 sites preferred natural images with strong and straighter edges more so than IT sites (Fig. 1b**;** comparing the median V1/V2 and IT site responses, values were 0.63 ± 0.02 vs. 0.61 ± 0.02 [monkey A] and 0.66 ± 0.02 vs. 0.62 ± 0.02 [monkey B], *P* = 0.017 and 0.0014, per an untailed Wilcoxon rank-sum test, with simple-difference = 0.08 and 0.10; num. of images across tests, 268–736).

**Table 2:** Selectivity to semantic features in both animals, per visual area.

	% observed (99th %ile of null distribution)							
Monkey A								
PIT	Primate, Rhesus macaque, Vertebrate 0.063 (005–0.007)		Face: 0.060 (0.005)	Head, Mammal, Skin: 0.057 (0.004–0.006)		Nose: 0.050, 0.005	Macaque: 0.047 (0.006)	Snout: 0.044 (0.004)
V4	Flower, Flowering plant, Groundcover, Herb, Subshrub: 0.031, (0.001–0.009)		Art: 0.023 (0.006)			Botany, Furniture, Leaf: 0.023 (0.003–0.005)		
	Plant: 0.031 (0.019)							
V1-V2	National Park: 0.057 (0.001)		Animal figure, Body of water, Botany, Flower, Flowering plant, Food, Geology, Geyser: 0.029 (0.001–0.009)					
	Plant: 0.057 (0.019)							
Monkey B								
PIT	Macaque, Mammal, Mouth,		Adaptation, Face, Snout: 0.029 (0.004–0.005)					Forehead: 0.022 (0.003)
	Primate, Rhesus macaque: 0.036 (0.005–0.007)							
	Vertebrate: 0.036 (0.007)							
V4	Red: 0.037, 0.002	Water: 0.031, 0.005	Building, Furniture, Table: 0.025 (0.003–0.005)		Barware, Cabinetry, Clothing, Dress, Drinkware: (0.019, 0.001)			
V1-V2	Line: 0.025, 0.019		Glass, Technology, Text: 0.019 (0.002–0.005)		Alarm clock, Barware, Camera accessory, Cameras & optics, Clock: 0.013 (0.001)			
	Water: 0.025, 0.005							

In summary, while we confirmed that neurons in early cortical regions were more modulated by Gabor patches and simple contours relative to more anterior cortical regions, these simple images were still not as effective in modulating responses as natural images. We also found that IT neurons showed strong preferences for images of primates, particularly macaques, including faces and bodies. Next, we sought to isolate the specific features that made neurons respond across these natural images.

### Neural response-guided generative algorithm

To identify the specific features encoded at each site, we used a generative neural network (“generator”) that creates images from lower-dimensional input vectors. The images contain many features that can combine to strongly evoke responses at each site. Thus, the images can serve as visualizations of the informational content encoded within the given site. This particular generator was trained to invert the activation patterns of CaffeNet layer fc6 units back to specific images^12^. It is distinct from conventional GANs (generative adversarial networks) which only train their generators to “fool” a discriminator network by randomly sampling throughout latent (input) space. While this generator was trained to do this as well, it was additionally trained on the input vectors corresponding to CaffeNet fc6-layer activations evoked by ImageNet images. Consequently, it is more expressive than conventional GANs because un-trained points in its input space may produce arbitrary visualizations that do not resemble any given object, in addition to an astronomical number of combinations of straight and curved contours, spots, colors and textures, many of which are reminiscent of objects and object parts in photographs^12,20^. We previously validated the use of this generator in a closed-loop approach for maximizing neuronal activity^21^.

In each experiment, first we selected one specific cortical site in the array, then identified the most likely location of its receptive field (RF) center (Supplementary Fig. 1, Methods). This served as the location on which to center the textures during the synthesis experiments. Synthetic images were 2°–3° in width across most experiments, larger than the conventionally accepted size of V1, V2, and V4 receptive fields near the fovea^13,14,22^; this allowed for contextual influences from outside the classical receptive field (e.g., surround suppression/facilitation) and avoided stimulus-edge artifactual responses. At the start of these experiments, we initialized the image generator with 30 randomly selected input vectors and displayed each of the 30 resultant images to the monkey, while recording spike rates from the site. Spike rates and input vectors were fed into an adaptive search algorithm (“covariance matrix adaptation evolutionary strategy” or CMA-ES)^11,23^ which found new candidate vectors likely to increase firing rates. The new vectors were given as inputs to the generator to start the next cycle of image generation (Fig. 1d).

We conducted over 250 experiments across both animals involving visually responsive channels (127 in monkey A; 125 experiments in B). The optimizing objective of each image-synthesis experiment was to increase the firing rate of the neuron(s) in the site —in virtually all visual neuroscience experiments, finding images that elicit strong activity is a foundational approach to functional interpretation. Most image-synthesis experiments resulted from a set of final-generation images that strongly activated the targeted site over controls. An evolution experiment was deemed successful if the site’s mean firing-rate response to the final cohort of images significantly exceeded two control image sets: (i) the reference images, which had been pre-selected to evoke strong responses from the site under study (*P* < 0.01, Wilcoxon rank-sum test) and to (ii) the first cohort/generation of synthetic images (see Methods). We found that the percentage of experiments that were successful per the bootstrap approach were 96.6% [monkey A] and 91.4% [monkey B] in V1/V2, 85.7% and 93.3% in V4, and 71.4% and 83.3% in IT, *N* = 28–70 experiments per area/monkey: Fig. 1e, f, Table 3). The final synthetic images often elicited more activity than the reference images, but less frequently so, suggesting that the reference images were well-chosen (% experiments resulting in images evoking stronger activity than references were 96.6% [monkey A] and 88.6% [monkey B] in V1/V2, 78.6% and 66.7% in V4, and 61.4% and 55.0% in IT, Table 3). Overall, we conclude that this method was effective throughout the ventral stream.

**Table 3:** Prototype synthesis experiments.

	V1	V4	IT
Monkey A			
Median change in firing rate to adaptive synthetic images (no. experiments)	36.5 ± 9.0 (29)	24.8 ± 7.5 (28)	16.2 ± 4.1 (70)
Median change in firing rate to fixed reference images	−2.0 ± 2.1	−3.1 ± 1.6	−2.3 ± 0.6
Percent of successful experiments, defined as those where the final synthesized images evoked higher responses than the first-generation images (95% CI of difference not including zero)	96.6	85.7	71.4
Percent of successful experiments, defined as those where the final synthesized images evoked higher responses than the reference images (Wilcoxon rank-sum test, one-sided, *P* < 0.02)	96.6	78.6	61.4
No. generations needed to reach half-maximum mean response (Wilcoxon rank-sum test, two-sided, contrasting V1/V2 and IT)	10.5 ± 1.0	13.7 ± 1.1	17.5 ± 2.2 *P* = 1.6 × 10^−33^, Z-value 12.1, *N* = 99 experiments
Monkey B			
Median change in firing rate to adaptive synthetic images (N experiments)	54.9 ± 15.7 (35)	46.4 ± 6.3 (30)	50.5 ± 11.6 (60)
Median change in firing rate to fixed reference images	−5.9 ± 1.6	−4.8 ± 4.1	−6.7 ± 1.3
Percent of successful experiments, defined as those where the final synthesized images evoked higher responses than the first-generation images (95% CI of difference not including zero)	91.4	93.3	83.3
Percent of successful experiments, defined as those where the final synthesized images evoked higher responses than the reference images (Wilcoxon rank-sum test, one-sided, *P* < 0.02)	88.6	66.7	55.0
No. generations needed to reach half-maximum mean response (Wilcoxon rank-sum test, two-sided, contrasting V1/V2 and IT)	12.0 ± 2.6	14.6 ± 2.7	16.4 ± 1.7
			*P* = 4.8 × 10^−33^, Z-value 12.0, *N* = 95 experiments

For brevity, we will refer to these synthesized images as “prototypes” — an allusion to prototype theory, which developed the idea that complicated concepts may be summarized by abstract templates^24^. Because neuronal selectivity becomes increasingly complex over the occipito-temporal pathway^25^, prototypes serve as pictorial descriptions of the specific types of colors, shapes, and textures encoded at each given site.

### Prototype differences across areas

**Fig. 2:**
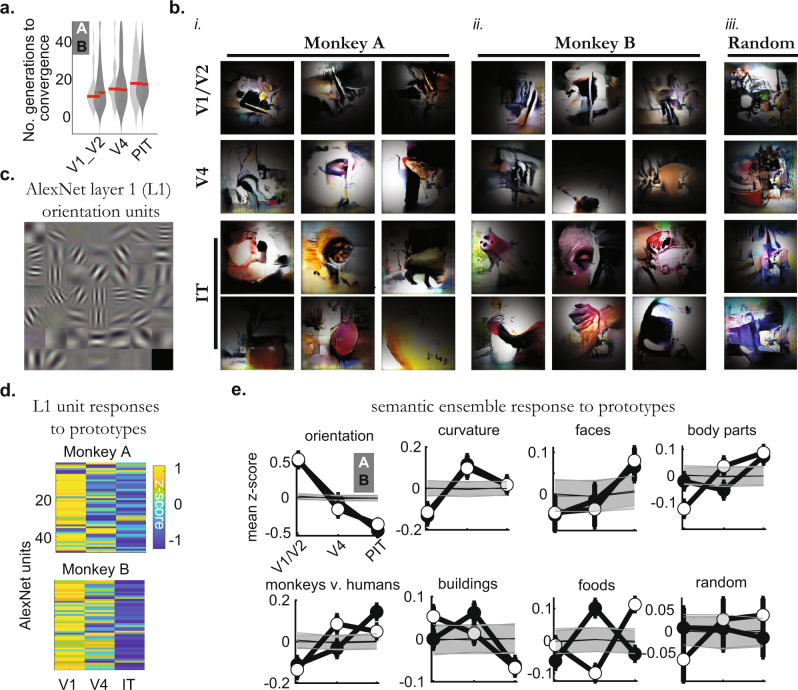
Prototype development and interpretability. **a** Number of generations needed to reach half of the final mean rate, as a function of the cortical region for both animals (A, B, black and white; red line: median, *n* = individual experiments per region, monkey A, V1/V2, 29; V4, 28; IT, 70; monkey B, V1/V2; 35, V4; 30 IT, 60). **b** Example synthetic images per monkey (*i-ii*) and visual area (rows), as well as examples of images synthesized during control random drift experiments (lacking neuronal guidance, *iii*). Masks show estimates of each site’s receptive field center. **c** Semantic ensemble for black-white contours consisting of 48 filters in AlexNet convolutional layer 1 (L1). **d** Mean activation levels of L1 units to all prototypes extracted from V1/V2, V4, and IT, per monkey. Colors represent the z-score across units and layers per monkey. **e** Different semantic ensemble responses to the extracted prototypes (black, monkey B, white, monkey A), using different populations of AlexNet fc6 units selected based on their responses to separate image sets (mean ± SEM; orientation, *n* = hidden units; orientation, 48; curvature, 249, faces, 33; body parts, 525; monkeys vs. humans, 309; buildings, 910; foods, 823; random, 200). Source data are provided as a Source Data file.

V1/V2 sites required fewer cycles to create their prototypes compared to IT sites, as defined by the number of generations needed to reach one half of the maximum response (median no. of generations were 10.5 ± 1.0 [monkey A] and 12.0 ± 2.6 [monkey B] for V1/V2 sites, in contrast, it was 17.5 ± 2.2 and 16.4 ± 1.7 for IT sites, these values were different between areas; *P* = 1.6 × 10^−33^ and 4.8 × 10^−33^, untailed Wilcoxon rank-sum test, simple-difference = 0.97, 0.99, *N* = 99, 95 experiments, Table 3, Fig. 2a, Methods). We interpreted these differences in convergence rates as the result of an increase in feature specificity^26^ as predicted by the hierarchical depth and as measured using an image-correlation-based technique (Supplementary Fig. 2). To substantiate this interpretation further, we conducted in silico generation experiments using units across AlexNet and found that units in each subsequent layer took more iterations to converge than those in the previous layer (Supplementary Fig. 2).

By visual inspection, the clearest difference in prototypes across areas was that V1/V2 sites often gave rise to achromatic contours, whereas IT sites gave rise to features reminiscent of faces, bodies, and fruits (e.g., rounded pink contours surrounding eye-like dark spots; long and smoothly curved black contours as in body parts; red and yellow contours as in apples and bananas; Fig. 2b). V4 sites often synthesized features intermediate to both V1 and IT, with some gratings and comma-shaped objects less reminiscent of specific object classes. In order to quantify differences across areas, we tested if prototypes can be reconciled with established views of neuronal selectivity in V1, V2, V4, and IT — for example, differences in selectivity for straight contours, curvature, and more complex object attributes. Our method leveraged AlexNet to test multiple hypotheses of interest, using “semantic ensembles” of artificial neurons. We defined a semantic ensemble as a collection of CNN units that are selective for a given visual attribute. For example, to test if V1/V2 prototypes contained more achromatic stripes than IT prototypes did, we first identified a set of AlexNet convolutional filters (units) trained to detect achromatic gratings (found in convolutional layer 1, 48 out of 96 units, Methods, Fig. 2c), independently of the prototype data. These specific units form the “achromatic-gratings ensemble”. We propagated all biological prototypes into AlexNet and examined the activation of the achromatic-gratings ensemble to each prototype. This is akin to an fMRI-analysis localizer technique^27^ where one selects voxels using one set of trials and tests the same voxels with new experiments (Supplementary Fig. 3). We found that the achromatic-gratings ensemble responded more strongly to V1/V2 prototypes than to V4 or IT prototypes (Fig. 2d, e). This is consistent with the hypothesis (derived from conventional stimulus paradigms) that these early prototypes contained more Gabor-like features.

Next, we used this flexible approach to test which area’s prototypes contained more curved contours, creating an ensemble of AlexNet layer fc6 units that preferred curved over straight Gabor patches; we subsequently found that this ensemble responded more strongly to V4- than to V1/V2- or IT prototypes (Fig. 2e; Supplementary Fig. 3, Methods); in contrast, face-preferring ensembles responded more strongly to IT prototypes than to V1/V2- or V4 prototypes — similar results were seen with body-preferring ensembles, and semantic ensembles sensitive to shape differences between monkeys and humans (Fig. 2e, Table 4). Thus, we show that many prototypes along the monkey visual recognition pathway begin with white/black contours, acquire curvature through V4, and later represent features reliably present in primates and animate objects.

**Table 4:** Prototype interpretation via semantic ensembles.

AlexNet ReLU6 units	Response to prototypes (z-scored, mean ± SE)			*P*-value (one-way ANOVA, cortical area as factor, F-statistic)
	V1/V2	V4	IT	
Monkey A				
Orientation ensemble (*N* = 48 units, AlexNet ReLu1)	0.54 ± 0.10	−0.16 ± 0.10	−0.37 ± 0.06	1.3 × 10^−11^, F = 30.1
Curvature ensemble (*N* = 249 units)	−0.12 ± 0.04	0.10 ± 0.04	0.02 ± 0.03	9.2 × 10^−04^, F = 7.1
Color ensemble (*N* = 329 units)	0.07 ± 0.04	−0.13 ± 0.04	0.05 ± 0.03	1.8 × 10^−04^, F = 8.7
Face ensemble (*N* = 331 units)	−0.04 ± 0.04	−0.03 ± 0.04	0.07 ± 0.03	5.2 × 10^−02^, F = 3.0
Body parts ensemble (*N* = 525)	−0.12 ± 0.03	0.04 ± 0.03	0.09 ± 0.02	4.0 × 10^−07^, F = 14.9
Animate ensemble (*N* = 187)	−0.11 ± 0.05	0.05 ± 0.05	0.06 ± 0.04	2.9 × 10^−02^, F = 3.6
Monkey v. Human ensemble (*N* = 309)	−0.14 ± 0.04	0.09 ± 0.04	0.05 ± 0.03	4.2 × 10^−05^, F = 10.2
Food ensemble (*N* = 823)	−0.01 ± 0.02	−0.10 ± 0.02	0.11 ± 0.02	3.6 × 10^−10^, F = 21.9
Buildings ensemble (*N* = 910)	0.05 ± 0.02	0.01 ± 0.02	−0.07 ± 0.02	4.5 × 10^−04^, F = 7.7
Random groupings ensemble (*N* = 200)	−0.06 ± 0.05	−0.02 ± 0.05	0.08 ± 0.04	9.0 × 10^−02^, F = 2.4
Monkey B				
Orientation ensemble (*N* = 48 units, AlexNet ReLu1)	0.51 ± 0.08	−0.05 ± 0.09	−0.47 ± 0.07	1.9 × 10^−13^, F = 36.3
Curvature ensemble (*N* = 249 units)	−0.13 ± 0.04	0.11 ± 0.04	0.02 ± 0.04	1.5 × 10^−04^, F = 8.9
Color ensemble (*N* = 329 units)	−0.02 ± 0.04	−0.01 ± 0.04	0.03 ± 0.03	0.65, F = 0.4
Face ensemble (*N* = 331 units)	−0.05 ± 0.04	−0.02 ± 0.04	0.07 ± 0.03	6.6 × 10^−02^, F = 2.7
Body parts ensemble (*N* = 525)	−0.02 ± 0.03	−0.05 ± 0.03	0.07 ± 0.03	5.5 × 10^−03^, F = 5.2
Animate ensemble (*N* = 187)	−0.12 ± 0.05	−0.09 ± 0.05	0.22 ± 0.04	1.3 × 10^−07^, F = 16.3
Monkey v. Human ensemble (*N* = 309)	−0.11 ± 0.04	−0.03 ± 0.04	0.15 ± 0.03	1.6 × 10^−06^, F = 13.5
Food ensemble (*N* = 823)	−0.06 ± 0.02	0.10 ± 0.02	−0.04 ± 0.02	3.5 × 10^−07^, F = 15.0
Buildings ensemble (*N* = 910)	0.00 ± 0.02	0.06 ± 0.02	−0.06 ± 0.02	2.8 × 10^−04^, F = 8.2
Random groupings ensemble (*N* = 200)	−0.08 ± 0.05	0.06 ± 0.05	0.02 ± 0.04	8.3 × 10^−02^, F = 2.5

These ad hoc tests were not meant to be comprehensive. Instead, their purpose was to address potential concerns that neuron-guided image synthesis is not comparable to the decades of previous findings in visual neuroscience, because the resulting images are too complex. Although we believe this image information density does reflect an intrinsic feature of neuronal selectivity (see next section), our semantic ensemble analysis provides two ameliorating conclusions. First, neuron-guided image synthesis could produce any of an astronomical number of images, yet analysis of prototypes incorporated previous notions of visual neuron selectivity across the ventral stream — with V1 neurons showing high activity to straight contours, and V4 neurons encoding curvature information. This consistency bolsters the credibility of the technique. Second, not only can we make useful comparisons, but we could also extract surprising insights, for example, showing that conspecific features are more reliably represented in IT than other features key to survival, such as the appearance of foodstuffs. Thus, here we demonstrate that neural prototypes are rich in information, and can be used towards multiple decoding purposes, as expected from a flexible neural code.

### Prototype complexity is comparable to segmented object parts

**Fig. 3:**
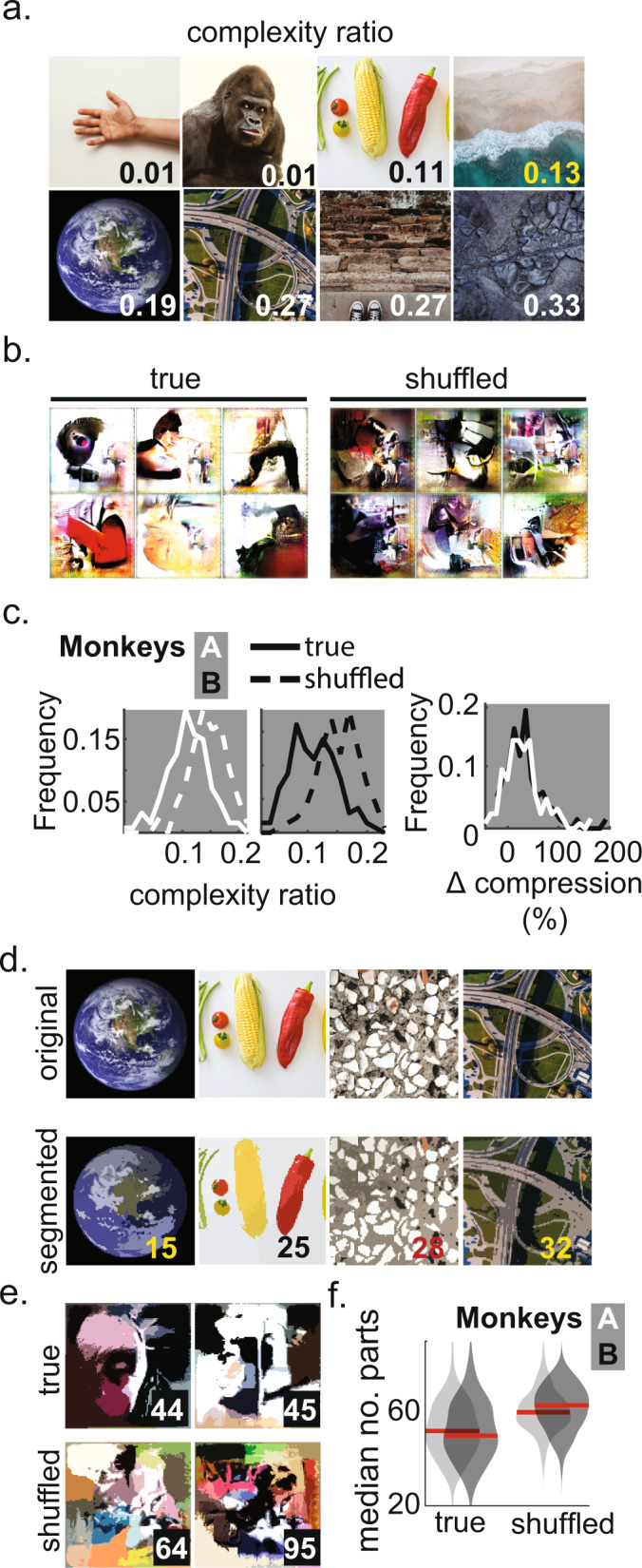
Compressibility of prototypes. **a** Examples of natural images and their complexity ratio annotated on the image (colored for visibility). **b** Examples of prototypes and random generator images (“shuffled prototype surrogates”). **c** (left, center) Ratio distributions for true and shuffled prototypes (solid, broken lines) per animal (black and white). (right) Percent difference in ratio value ([shuffled ratio–observed ratio]/observed ratio × 100%) for both animals (black and white). **d** Examples of natural images and their parts per the mean-shift segmentation algorithm. The number of segments annotated on the image. **e** Example segmentations of prototypes and surrogates (with annotations as in panel **e**). **f** Median number of segmentation parts for true and shuffled (± SE), for both monkeys (black and white, *n* = prototypes, 127, 137). Source data are provided as a Source Data file.

We observed that neurons generally produced patterns of specific shapes, colors, and textures that looked like background-segmented objects; prototypes appeared less chaotic, less spread-out, and less texture-like than random samples synthesized by the generator or than samples resulting from unsuccessful evolution experiments. To quantify this observation, we measured image complexity using an image decomposition operation called the discrete cosine transform^28^. This operation transforms a given image into a set of coefficients, corresponding to cosine component functions with different frequencies. By keeping the fewest components (i.e., fewest non-zero coefficients) necessary to reconstruct the image, the image is “compressed”. The *reconstruction complexity* of an image can be measured as the ratio of the minimum number of components needed to reconstruct the image divided by the total original number of components. This is the inverse of the commonly reported *compression ratio* and provides a measure of image complexity, and so we call it a *complexity ratio*. More complex images are harder to compress and vice versa, a notion of complexity based on minimum description length^29^ used in physics, math, and computer science. First, we measured the complexity ratio for various natural images and confirmed that its value was lower for photographs containing simple, background-segmented objects; for example, images depicting hands over white backgrounds were less complex than cluttered images such as aerial views of a city (Fig. 3a). Next, we set out to measure the complexity of prototypes. We found that neurons appeared to titrate prototype complexity: all experiments began with a first generation of images containing monochrome irregular textures with a median complexity ratio of 0.033 ± 0.004, yet prototypes converged to higher complexity values — the median complexity ratio values for prototypes were 0.161 ± 0.005 (monkey A) and 0.162 ± 0.006 (monkey B, ± SE). To measure the generator’s bias for complexity, we created surrogate “shuffled” prototypes by sampling the generator’s input space in two ways: a) by shuffling the order of elements in the input vectors obtained in the final generation of each evolution, and b) by simulating “random-evolution” experiments where the scores guiding the evolution were replaced by the inverse of a distance to a target random vector (having the same norm as each prototype vector; see Methods). We then compared the complexity ratio of the neuronally guided prototypes against the surrogate prototypes (Fig. 3b). We found that true prototypes were less complex than surrogate, shuffled prototypes, which showed a median complexity ratio of 0.224 ± 0.001 [monkey A] and 0.226 ± 0.001 [B] (an untailed Wilcoxon signed rank test contrasting true vs. shuffled (per monkey) yielded *P*-values not exceeding 2.5 × 10^−22^, with simple difference = −0.3, −0.2). Random evolution surrogates had a median of (0.196 ± 0.003 [monkey A], 0.204 ± 0.005 [monkey B]; *P*-values not exceeding 4.3 × 10^−14^, simple-difference = 0.22 and 0.23, *N* = 127, 137, Fig. 3c). Overall, neuronally guided images were 25-28% less complex (i.e., more compressible) than the surrogate samples from the generator’s input space (Fig. 3c, rightmost panel). Thus, neurons were not simply driving up complexity to the limits of the generator, nor were they suppressing it to the generator’s minimum capability.

There were no systematic differences in median complexity ratios across visual areas (0.159 [monkey A], 0.174 [monkey B], *P* = 0.07 and 0.90, Kruskal-Wallis test with site area as main factor), which was not surprising because the synthetic images were not resized precisely to each site’s receptive field, only aligned to their estimated centers (Supplementary Fig. 2). Further, since most experiments involved multi-unit signals, the complexity of prototypes often reflected the information of multiple receptive field centers and surrounds.

We established a context for these results with comparisons to classic stimulus sets, real-world photographs and to AlexNet hidden unit visualizations (which under our hypothesis can also be referred to as “prototypes”). First, we measured the complexity ratio of images from iconic stimulus sets frequently used to characterize tuning in V1, V2, V4 and posterior IT, such as Gabor patches, gratings, line shapes and bounded curved objects^30–32^, and found that their median complexity ratios were 0.001 ± 0.001 (Gabor patches), 0.010 ± 0.001 (Cartesian, hyperbolic gratings, line shapes) and 0.033 ± 0.001 (curved contour objects). Thus, traditional stimulus sets may be too simplistic to access the upper limits of a neuron(s) range of firing.

Next, we analyzed the complexity of real-world photographs from a sample of randomly selected images from ImageNet. These photographs yielded a median complexity ratio of 0.4885 ± 0.0012 (we sampled 40 images from each ImageNet category, *N* = 40,000 total). Neuronal prototypes were much less complex: the complexity ratios for monkey A prototypes yielded a median rank of the 5.6th percentile of photographs, while for monkey B it was 5.7th percentile (Wilcoxon signed rank test comparison to ImageNet complexity ratios: *P* = 1.33 × 10^−66^ and *Z* = −17.24 for monkey A, with *P* = 2.95 × 10^−71^ and *Z* = −17.85 for monkey B). These results suggest that neurons have limits to the complexity they can encode; neuronal prototypes contain less information than typical photographs (consistent with views of efficient coding) but prefer more information than typically present in classic stimulus sets.

Lastly, we measured the complexity ratio of prototypes evolved from AlexNet units and found they were higher than those of the biological prototypes (Supplementary Fig. 2; AlexNet prototype median complexity ratio of 0.209 ± 0.003 per 218 units sampled across layers). Thus, AlexNet is likely sensitive to features that are only partially preferred by neurons of the ventral stream.

Another way to quantify the relative complexity of any given prototype is to count its number of distinct parts. We used a segmentation algorithm called mean-shift clustering^33,34^ to estimate the number of pixel groups present in an image: the algorithm found fewer parts for natural images containing single- and multiple objects, and more parts for cluttered images (Fig. 3d, e). The true neural prototypes had fewer segments than both shuffled- and random-evolution prototypes (median no. of parts for true prototypes were 52.1 ± 1.1 [monkey A] and 50.8 ± 1.7 [monkey B]; for shuffled- and random-evolution prototypes, values were 60.0 ± 1.6 [monkey A] and 62.6 ± 1.8, [monkey B], *P* values not exceeding 1.8 × 10^−6^, Wilcoxon signed-rank test contrasting true vs. shuffled, per monkey, simple differences –0.188 and –0.186, *N* = 127, 137; Fig. 3f). As above, we also applied the algorithm to previously published stimulus sets and found that the median number of parts present in Gabor patches, gratings, line shapes and curved contour objects ranged from 12.9 ± 0.9 to 20.1 ± 0.3.

In summary, we found that cortical neurons led to the synthesis of images that were less complex and comprised fewer parts than those drawn randomly from generative input space and much less complex than full scenes (ImageNet photographs). However, these synthetic images were also more complex than the simpler visual stimuli typically used to characterize tuning, such as Gabor patches or flat solid curved shapes. Overall, this suggests that the neural code for primate object recognition represents features that are less complex than full scenes (i.e., scene/object parts), but still contain more information than is captured by artificial contour-based stimuli.

### Prototypes are associated with salient features in natural scenes

Per some theories of efficient coding, the visual system should abstract information that relates directly to behavior^8^. By visual inspection and per the semantic ensemble analyses, many of the prototypes we extracted resembled motifs present in photographs of animal faces. Macaque monkeys are particularly social animals that obtain important cues from the facial expressions of conspecifics^35–37^ and spontaneously observe faces, objects that look like faces^38^, and monkeys/animals in general^35^. We hypothesized that the prototypes obtained in the previous experiments might correspond to image regions more likely to be foveated during free viewing (a relationship predicted by theories of information selection in V1, and extended here along the ventral stream^10^). Such image regions are highly salient, as defined by low-level cues^39,40^ and by cognitive information (e.g. social features).

**Fig. 4:**
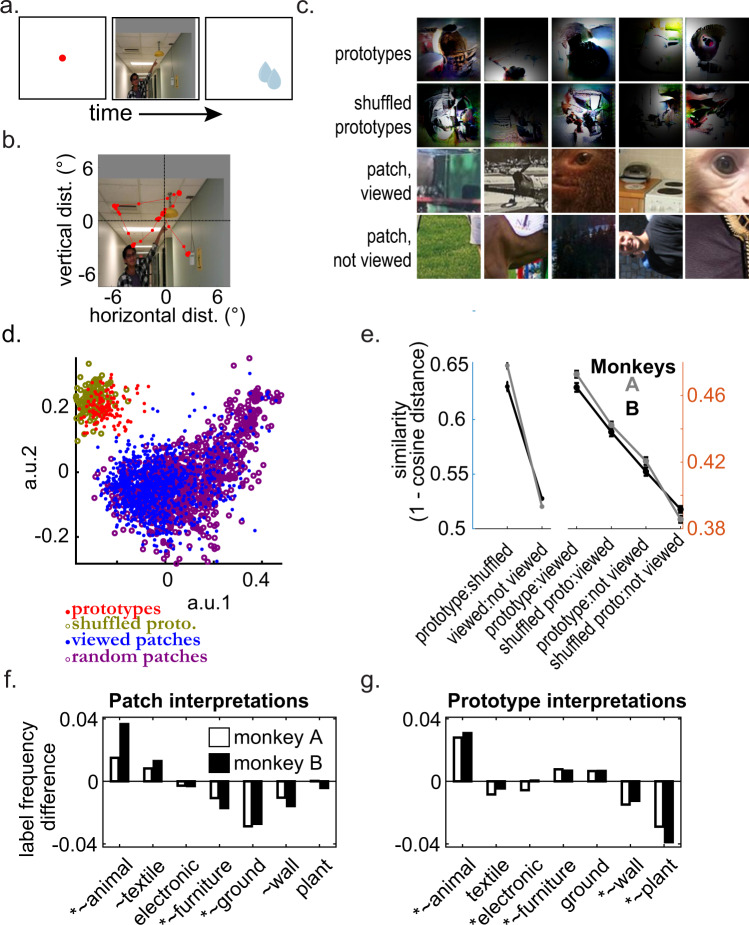
Evidence for active encoding based on free viewing. **a** Schematic of free viewing task flow. **b** Example trial of animal A’s eye-tracked path, beginning at fixation (0,0)° and making ~5 saccades (red dots/line). **c** Example prototypes, shuffled prototype surrogates, scene patches viewed by the monkeys, and scene patches not viewed by the monkeys. Black masks on prototypes and shuffled prototype surrogates show an estimate of the location of the receptive field center. **d** Scatter plot representation of the image fragment types in **c** in AlexNet fc6 space (red = prototypes, gold = shuffled prototypes, blue = viewed patches, purple = non-viewed (random) patches). **e** Mean similarity of fc6 representations between image sets denoted on the horizontal axis. Gray for monkey A, black for monkey B. Left panel compares prototypes and viewed patches with their corresponding (same image type) random control. Right panel shows a similarity between image sets curated by viewing patterns on photos to synthesized images. Standard error bars are shown (*n* = distance values between prototypes and patches, 930,500 [125 observed/shuffled prototypes and at least 7444 viewed/non-viewed patches]) **f** Bar charts showing differences in the frequencies that certain COCO-stuff categories are identified between viewed and not-viewed patches. Positive values indicate a visual feature category is more common in viewed patches, negative values indicate a category is more common in not-viewed patches. Data for both monkeys is shown (white = monkey A, black, monkey B). “*” indicates a *χ*^*2*^ proportion test *p*-value < 1.9 × 10^-3^ (Bonferroni adjustment for *N* = 27) for monkey A, “~” indicates the same for monkey B. Only categories that are abnormally common in at least one of eight data groups are analyzed (see Methods). Viewed patches are enriched with animal-associated visual features while non-viewed patches are enriched in visual features associated with furniture, ground, and walls. COCO-stuff has two “furniture” groups, only the “stuff” furniture group met inclusion criterion. **g** As in panel f, except showing differences between true and shuffled prototypes. Positive values indicate a category is more common in true prototypes. True prototypes are enriched in visual features associated with animals and furniture, while shuffled prototypes are enriched in features associated with plants (not food-related) and walls. Source data are provided as a Source Data file. Depicted individuals have consented for publication, and have seen these photos in context of the publication.

To test whether prototypes were related to regions of saliency, we presented the animals with 5223 photographs of natural scenes; the set included animals in natural settings, human-made environments (i.e., cityscapes), artificial objects, and photographs of their own holding-facility environment (Methods). During each session, the animals began a given trial by acquiring fixation on a spot at the center of the monitor, and after ~100 ms a large photograph appeared, replacing the fixation point (Fig. 4a). Images were displayed for 1000 ms, and subjects could look anywhere within the area of the monitor to get reward. The monkeys made up to 5 saccades per second (Fig. 4b). For each image, we cropped a macaque-fovea-sized 3° × 3° (width x height) “scene patch” corresponding to the first acquired fixation^41^. All patches were compared to the prototypes in AlexNet fc6 space using (1 – cosine-distance) as a measure of similarity (i.e., cosine similarity).

Because the generator images are not photorealistic, we predicted that the mean distance between prototypes and natural image fragments would have an arbitrary non-zero value. Therefore, we needed other distances for comparison. We measured the mean distance between shuffled prototypes (see previous section) and the scene patches (Fig. 4c), as well as the mean distance between prototypes (true and shuffled) and random scene patches. Random scene patches were obtained by randomly substituting the images that elicited the monkeys’ eye movements and extracting the new regions marked by the same fixation patterns.

The comparisons across image set types were the most informative. We found that prototypes and viewed patches showed the highest similarity (median of 0.47 ± 2.0e^−03^ [A] and 0.46 ± 2.6e^−03^ [B]), followed by shuffled prototypes to viewed patches (0.44 ± 2.0e^−03^ [A] and 0.44 ± 2.2e^−03^ [B]), prototypes to non-viewed patches (0.42 ± 2.3e^−03^ [A], 0.41 ± 2.5e^−03^ [B],) and finally, the lowest similarity was between shuffled prototypes and non-viewed patches (0.39 ± 2.0e^−03^ [A], 0.39 ± 2.0e^−03^ [B]). Overall, the mean cosine similarity between generator images and photographic patches was 0.429 ± 0.002 (for monkey A) and 0.423 ± 0.002 (monkey B); Comparisons within image set types established a baseline; we found the overall patch-to-patch similarity was 0.520 ± 4 × 10^−4^ (A) and 0.528 ± 4 × 10^−4^ (B), and the overall generator image-to-image similarity, 0.649 ± 0.003 (A) and 0.642 ± 0.005 (B). Thus, we found that across group similarities are lower than within group similarities, but that the highest across-group similarity was between viewed patches and true prototypes. This effect was reliable across animals — and remarkable considering the comparisons were based on fewer than ~137 prototypes per animal, using an untrained natural behavior.

We then tested if it was coincidental that prototypes were closer to viewed patches than shuffled prototypes. We constructed a null distribution via permutation testing, by sampling two groups of AlexNet fc6 vectors at random from the combined set of true and shuffled prototypes, then measuring how much closer one random group was to viewed patches compared to how much closer the other group was to the same viewed patches. We repeated this 499 times. We found (in both animals) that less than 0.2% of random samples met or exceeded our experimental evidence for how much closer true prototypes are to viewed patches than shuffled prototypes. We conclude that prototypes were more similar to scene regions spontaneously fixated by the animal, compared to shuffled prototypes; this is consistent with the hypothesis that prototypes contain features of behavioral significance.

We have shown that viewed patches were closer to true prototypes than to shuffled prototypes, and also that true prototypes were closer to viewed patches than not-viewed patches. Next, we set out to test whether this effect was due to differences in the behaviorally relevant content of images or due to low-level image properties. Specifically, by low-level properties, we mean those which can be calculated globally (i.e., an expected value of a function of all pixels), termed global image statistics. As we will show, our decision to shuffle the order of input vector elements changed the behaviorally relevant content of images more than the global image statistics such as textural properties (which are less directly behaviorally relevant). This analysis evinced agreement in the cognitive information content of viewed patches and true prototypes.

In Supplementary Fig. 4, we show that viewed and not-viewed patches had a greater disparity in global image statistics than true and shuffled prototypes. The image statistics we used were energy, entropy, stationarity, mean luminance, and Tamura’s textures^42^; all were calculated on the L* channel of the CIE 1976 L*a*b* color space. Median values are reported in Supplementary Table 1. We pairwise-subtracted the values for all shuffled prototypes from all true prototypes and did the same for viewed and not-viewed patches. The values in the “surrogate” group (shuffled prototypes or not-viewed patches) could have been systematically offset, systematically more variable (greater dispersion) or both. The median pairwise signed difference determined the offset (reported in Supplementary Table 2 and Supplementary Fig. 4). The unsigned difference combined shift and spread, and pairwise dividing out the “baseline” group (true prototypes or viewed patches) scaled the difference by a baseline (the fractional absolute pairwise distance). Both prototypes and patches showed significant shifts in all statistics for both monkeys except for the directionality of prototypes and the regularity of patches, (untailed Wilcoxon ranksum test, all significant *P-*values <5.9 × 10^−9^, all non-signficant values > 0.1, Supplementary Table 2). The fractional absolute pairwise difference showed that patches evinced more shift and spread because all simple-difference formula effect sizes were positive except for Monkey B’s entropy statistic (Wilcoxon ranksum test, untailed, all *P*-values zero to single precision; Supplementary Table 3). This confirmed that true and shuffled prototypes were comparatively more similar than viewed and not-viewed patches according to global image statistics, corroborating the true/shuffled and viewed/not-viewed columns of Fig. 4e.

Next, we assessed the content of patches and prototypes by associating images with semantic categories (Fig. 4f-g). Our goal was to quantify the relative frequency of key terms associated with each group. Despite our previous use of Google Cloud Vision above, it was not appropriate for this analysis because its vocabulary and verbosity were too large. Thus individual words were too infrequent to compare relative frequencies across image sets and there was no easy way to group words or captions into semantic categories. Hence, we adapted a ResNet architecture trained for semantic segmentation on the COCO-Stuff dataset^43^, which augments the COCO dataset with additional labels and groups 182 possible labels into 27 semantic categories (see Methods for details and architecture, Supplementary Table 4 for label text and hierarchy). We then assessed the frequency that a semantic label category was associated with images in each of the eight image datasets (two monkeys and four partitions: patches, not-viewed patches, true prototypes, shuffled prototypes). We restricted the analysis to the seven semantic categories that were assigned more than a minimum number of times, (see Fig. 4d, Method, Supplementary Table 5 for values). The occurrence frequencies for these categories were then subjected to a *χ*^2^ proportion test with significance level set to 0.05/27 (Bonferroni adjusted for *N* = 27) to test whether a given category was more common in viewed or non-viewed patches or true or shuffled prototypes (Supplementary Table 6). The analysis confirmed there were significant differences between the content of true and shuffled prototypes, and that these were of similar magnitude and character to those between viewed and not-viewed patches. Namely, true prototypes and viewed prototypes were strongly associated with animals, and shuffled prototypes and not-viewed patches were respectively associated with background and non-monkey-food plants (see below). Recalling that true and shuffled prototypes were not as different as viewed- and not-viewed patches according to global image statistics, it was noteworthy that in the case of image content, they did have similar differences. We conclude that shuffling the order of input vector elements affected semantic content more than global image statistics, and thus shuffling was an acceptable way to produce non-trivial surrogates for image content.

More importantly, assessing the semantic content of patches and prototypes also bolsters the relevance of ethological and cognitive information to neural representations, while also revealing why, in human terms, viewed and non-viewed patches differed according to global image statistics. We found that viewed and not-viewed patches showed a strong foreground-vs.-background dichotomy, with not-viewed patches being more frequently associated with background-type categories. Specifically, *Ground* (*Χ*^*2*^ = 49.2, *p* = 2.3 × 10^−12^ for monkey A, *Χ*^*2*^ = 43.4, *p* = 4.4 × 10^−11^ for monkey B) and *Wall* (*Χ*^*2*^ = 7.9, *p* = 4.8 × 10^−3^ for monkey A, *Χ*^*2*^ = 21.2, *p* = 4.1×10^-6^ for monkey B, e.g., *Ground* included *gravel*, *mud*, *railroad*; *Wall* included *brick*, *panel*, *wood*, etc.). Since backgrounds like grass and walls are plain and foreground objects tend not to be, this explains the difference in global image statistics. Conversely, we found that true and shuffled prototypes showed an animal-vs.-plant dichotomy, with shuffled prototypes being more frequently associated with plants (which were not food-related for monkeys). Association with homogenous features was unlikely for shuffled prototypes since global image statistics were preserved. Consequently, the ResNet model associated the heterogeneity of shuffled prototypes with the *Plant* category. Thus, *Plant* was the null/baseline category for the image generator. *Plant* labels were the most common in shuffled prototypes (*Χ*^*2*^ = 37.2, *p* = 1.1 × 10^−9^ for monkey A, *Χ*^*2*^ = 73, *p* = 1.3 × 10^−17^ for monkey B) — specifically, these were non-monkey-edible plants (e.g., *tree*, *grass*, *flower*). A background category weakly associated with shuffled prototypes is *Wall* (*Χ*^*2*^ = 42.6, *p* = 6.8 × 10^−11^ for monkey A, *Χ*^*2*^ = 22.7, *p* = 1.9 × 10^−6^ for monkey B). The most important result was that both viewed patches and true prototypes were more likely to be assigned a label in the *Animal* category (e.g. *bird*, *cat*, *elephant*, etc.) than non-viewed patches (*Χ*^*2*^ = 20.0, *p* = 7.7 × 10^−6^ for monkey A, *Χ*^*2*^ = 50.2, *p* = 1.4 × 10^−12^ for monkey B) or shuffled prototypes (*Χ*^*2*^ = 46.9, *p* = 7.4 × 10^−12^ for monkey A, *Χ*^*2*^ = 17.3, *p* = 3.2 × 10^−5^ for monkey B). Thus, true prototypes were independently and robustly confirmed to have more animal relevance (and therefore behavioral relevance) than shuffled prototypes, and they also had content more similar to viewed patches.

The remaining features may also help illuminate the effects of neural guidance on image synthesis. *Furniture* was a feature that was moderately enriched in non-viewed patches (*Χ*^*2*^ = 34.5, *p* = 4.3 × 10^−9^ for monkey A, *Χ*^*2*^ = 32.4, *p* = 1.2 × 10^−8^ for monkey B) but also in true prototypes (*Χ*^*2*^ = 33.1, *p* = 8.9 × 10^−9^ for monkey A, *Χ*^*2*^ = 20.6, *p* = 5.5 × 10^−6^ for monkey B; specifically, “furniture < indoor < stuff” from Supplementary Table 4). *Furniture* was a category that contained items that might be behaviorally irrelevant (e.g., *stairs*); alternatively, it contains items in the monkey’s housing and lab environments (e.g., *cupboard*, *cabinet*), and those associated with specularity and/or strong luminance contrasts (e.g., *desk*, *mirror*, *light*) as might develop in V1/V2 prototypes. The remaining categories, *Textile* and *Electronics* were assigned frequently enough to meet the criterion for inclusion, but were inconsistent (i.e., did not occur with significantly different ratios for both monkeys, see Supplementary Table 6). From this semantic segmentation and labeling analysis, we can first conclude that shuffling prototypes destroyed the content of images more consistently than it modified global image statistics and overall textural properties, thus validating shuffling as a method for generating surrogates. Most importantly, we can conclude that viewed patches and true prototypes shared the important ecologically relevant property of being related to animals.

## Discussion

Neurons in visual cortex must encode specific information from retinal scenes, a process complicated by the fact that downstream neural systems may have to decode different types of information at different periods in the animal’s life. The code must be both rich and sufficiently flexible to allow various kinds of patterns to be decoded, but not so rich that it overfits incidental features of the visual world; to paraphrase, the code must be “as simple as possible, but not simpler”^44^. In this study, we defined features of the neural code for visual recognition as implemented in early and mid-level visual areas of the cortical hierarchy^45^, using an image-generating system, which when combined with a search algorithm, efficiently found images that maximized neuronal firing rates. We called these images *prototypes*. Our work contrasts with other studies referencing efficiency in neural coding because it does not attempt to characterize sparseness of the spiking code^46,47^, nor a mutual-information relationship between spiking patterns and image statistics. Instead, our concern with efficiency is image-based, following the classic motivation of identifying visual attributes that maximize “concentration of information,”^3^ using high firing rates as markers for these attributes.

Why use maximal firing rates as an optimizable objective? Under optimal conditions, neurons should use their full output capacity^48^: for example, in a rate-based code, this means sometimes firing at a low rate and at other times firing at a high rate. While almost any image stimulus set can modulate neuronal responses, the generator-based approach leads to images that explore more of a neuron’s dynamic range — at least as well as natural images. Although we are exploring alternative objectives, including population pattern responses, in terms of efficient coding, achieving a broad output range is a reasonable first step.

Prototypes were information-rich and helpful for alternative ad hoc coding strategies, consistent with previous findings that incidental visual information can be decoded from neuronal responses to complex images^49^. Although the generator produced abstract combinations of texture-like images, neurons generally guided the development of simpler, spatially constrained motifs of intermediate complexity^50^. This complexity was also below that of prototypes encoded by units in neural networks such as AlexNet (which have similar complexity to shuffled prototypes). The less-coherent conglomeration of textures in AlexNet and shuffled prototypes were less compressible than well-segmented object parts^51^. This is consistent with the finding that convolutional neural networks are biased towards texture detection (curiously, networks trained to be robust to adversarial attacks learn to represent shapes rather than textures^52^). Furthermore, there was a floor to the preferred level of complexity, as neurons moved towards a level of information density in their synthetic images that exceeded that of the initial generation of synthetic images and that of Gabor patches, curved contours, and gratings. The complexity of these prototypes is comparable to that of textured, segmented object parts. Previous studies showed that adding multiple objects within the classic receptive fields of individual neurons can lower their firing rate^53,54^, likely due to mechanisms such as normalization. Our study goes beyond these findings by showing these mechanisms can be engaged by response optimization, and by showing how such normalizing mechanisms relate to actual visual attributes. This conceptual advance also establishes an upper limit to the neurons’ representational complexity (e.g., preferred stimulus density). The observed titrated complexity of prototypes (and their foreground/background segmentation) also overlap with findings that images are more memorable when they are well-positioned, bright and free of clutter^55,56^.

Even though measured prototype complexity was comparable across visual areas, they encoded for different types of information. How do we reconcile these findings? First, we evolved using fixed-size textures that were often larger than a given neuron’s receptive field, so the neuron was free to operate on any portion of the texture — both through its classical receptive field and surround. Examining regions limited to classical receptive fields would affect the measured value of prototype complexity, so our results have to interpreted as an upper value. Still, in simulations using AlexNet, we found that units with very small receptive fields (11 x 11 pixels) that operated on larger textures (227 × 227 pixels) evolved images with higher complexity values than we observed in the neuronal data, suggesting that V1/V2 and V4 array sites operated over a larger fraction of the texture. In multiunit recordings, prototypes likely reflect the function of multiple local RF centers; in single-unit recordings, they will include the effects of surrounds and possibly longer-range horizontal projections^57^. Therefore, the prototypes may reflect an amalgam of simpler features, as expected if neighboring single neurons in multiunit signals had overlapping but not identical functional tuning. Also relevant is that our definition of complexity is based on the minimal description length^29^, a well-known principle that is distinct from some common usages in the neuroscience literature, such as referring to images that are composites of simpler images or to the complexity of an object being pictured rather than to the optical patterns of the image. Given this, there are several ways that neurons that individually encode for semantically simpler features could also yield prototypes with higher reconstruction complexity.

The visual system stores important motifs of the animal’s natural environment that serve as part of an internal model of the world. Given the constancy of the natural world and the importance of other animals for survival and reproduction, it is sensible that this internal model should subsume features related to generic regions of saliency^40^ and diagnostic of animals^58,59^, as well as the influence of natural scene statistics^6,50,60^. Our study advances these views by explicitly demonstrating the content and complexity of those features. Of note, the creation of specialized prototypes does not preclude identification of other, non-encoded objects: artificial neural networks show that any given filter (by rough analogy, any artificial “neuron”) can be used to analyze an image, even if the image does not contain any shapes that match the exact shape of the filter — as long as the subsequent decoder is well-trained and has access to other neurons. Put succinctly, motif specialization should make some objects easier to decode downstream but does not preclude performing generic classifications. In part, this is shown by our analysis that the same set of prototypes can be used to extract different kinds of information, for example, regarding orientation, curvature, and other semantic-category-level hypotheses, such as faces, body parts and foods. This is likely what the decoding neural circuits in different parts of the brain (e.g., prefrontal cortex, perirhinal cortex) must do, depending on the day-to-day needs of the animal.

## Methods

All procedures received ethical approval by the Washington University School of Medicine Institutional Animal Care and Use Committee and conformed to NIH guidelines provided in the Guide for the Care and Use of Laboratory Animals. All relevant ethical regulations for animal and non-human-primate testing and research were followed.

### Experimental setups

Experiments were run in two identical experimental rigs, each controlled by a computer running MonkeyLogic 2^61^. Stimuli were presented on ViewPixx monitors (ViewPixx Technologies, QC, Canada) at a resolution of 1920- × 1080 pixels (120 Hz, 61 cm diagonal); subjects were positioned 58 cm from the monitor during all experiments. Images were presented at the screens’ maximum resolution after rescaling to match the size of the image in degrees of the visual field. Image sizes generally were 3° per size (sometimes up to 4°) as needed to cover the receptive field and surround. Eye position was tracked using ISCAN infrared gaze tracking devices (ISCAN Inc., Woburn MA). For most experiments, the animals performed simple fixation tasks (i.e., holding their gaze on a 0.25°-diameter circle, within a ~1.5–2.5°-wide window on the center of the monitor for 2–3 s to obtain a drop of liquid reward (water or diluted fruit juice, depending on the subject’s preference). Rewards were delivered using a DARIS Control Module System (Crist Instruments, Hagerstown, MD).

### Neuronal profiles

Two male adult rhesus macaques (A and B, *Macaca mulatta*, ages, 10–11 kg) were implanted with chronic floating microelectrode arrays in the right hemisphere: one array at the posterior lip of the lunate sulcus (near the V1/V2 transition), one on the prelunate gyrus (V4) and another anterior to the inferior occipital sulcus (PIT). Based on standard anatomical descriptions^13,14,62^, we refer to these sites as V1/V2, V4, and IT. The locations of the arrays were chosen based on sulcal landmarks and on local vasculature, resulting in an unbiased sample of neurons. We use the term *site* to refer to both single- and multiunits; in all experiments, sites comprised mostly multiunits and some single units.

### Microelectrode arrays

Arrays were manufactured by Microprobes for Life Sciences (Gaithersburg, MD). Each array consisted of a ceramic base with 16–32 working electrodes (plus four reference/ground electrodes) made of platinum/iridium, with impedance values of 0.7–1.2 MΩ and lengths of 1.6–2.4 mm (4 mm for grounds and reference electrodes). To implant the arrays, a craniotomy was performed in the occipito-temporal skull region, followed by a durotomy that allowed visualization of sulcal and vasculature patterns; arrays were secured to a stereotaxic arm via suction and inserted into the cortical parenchyma at a rate of ~0.5 mm every three minutes, regulated by visual inspection of tissue dimpling. Arrays were fixed in place using titanium mesh (Bioplate, Los Angeles, CA) over collagen-based dural graft (DuraGen, Integra LifeSciences, Princeton NJ), and cables protected using Flexacryl (Lang Dental, Wheeling, Illinois). Omnetics connectors were housed in custom-made titanium pedestals (Crist Instrument Co., Hagerstown, MD).

### Data pre-processing

Data was recorded using Omniplex data acquisition systems (Plexon Inc., Dallas, Texas). At the start of every session, the arrays were connected to Omniplex digital head stages; all channels underwent spike auto-thresholding (incoming signals were recorded if they crossed a threshold amplitude value, determined within each channel, such as 2.75 standard deviations relative to zero-volt baseline). Manual sorting of units took place if the 2D principal component features of detected waveforms showed clearly separable clusters; clusters not overlapping with the main “hash” cluster were described as “single units” if they showed a refractory period; all other signals were described as “multiunits.” No further spike sorting was performed, as the closed-loop, real-time nature of our experiments made post-spike processing limited in usefulness.

### Localization of receptive field centers

To estimate the best retinotopic location for evolution experiments, the animals performed a fixation task while a 2°-wide image was flashed for 100 ms on randomly sampled locations within an invisible grid. On most days, the grid ranged from (−8,8)° in width to (−4,4)°, in steps of 1–2°. Monkey A participated in 39 receptive fields (RF) center mapping experiments, and monkey B in 51. Each position was tested an average of 6.5 ± 0.04 times (mean ± SE, *N* = 6906 total presentations across positions and experiments). The mean responses to each position were interpolated into a (100x100)-pixel image (*griddata.m*) and then fit using a 2-D Gaussian function (*fmgaussfilt.m*)^63^. Most neurons responded best to images placed peri-foveally, ranging from 0.3° to 3° in monkey A and 0.6° to 2.0° in monkey B, with V4 sites having a subset of particularly eccentric RFs in both animals (Supplementary Fig. 1). Fits showed increasingly larger Gaussian widths along with V1/V2, V4, and IT (Table 1), although they were too large to correspond to stricter definitions of “receptive fields.” These experiments were also used to select the sites for subsequent analyses based on their visual responsiveness. For each site, visual responsiveness was defined as the mean spike rate during the first 50–200 ms after image onset (the *late window)* minus the mean spike rate during the first 1–49 ms after image onset (*early window*). Across experiments, a given site was defined as *visually responsive* if its firing was modulated by image position in at least 10% of all receptive-field-mapping experiments (per one-way ANOVA, *P* < 0.01 after false discovery rate correction; the 10% threshold was chosen because some sites showed single-unit signals that appeared transiently for a few days, then disappeared).

### Selectivity

Neurons were tested for selectivity using a 754-image datastore. The datastore included photographs of animals (including humans and monkeys, either shown as a whole or segmented into face/body-part pictures), artificial objects (e.g., clocks, cars, kitchen utensils), places (both outdoors and indoors), and foods (fruits, vegetables). The set also contained computer-generated images such as Gabor patches (straight, curved, and radial), curved bounded stimuli^64^, and generator images from previous publications^21^ and from neural network simulations (sources included our own photographs, ImageNet 2012, and datastores accumulated over the years). To select the ImageNet images, we created a datastore object (*imageDatastore.m*) and sampled ~500 random images from it. Neuronal sites were defined as selective if their firing rate changed reliably upon presentation of different images (*P* < 0.001, one-way ANOVA run per site per experiment, with false-discovery rate correction; for monkey A, the median number of unique images per experiment was 318.3 ± 17.1, presented an average of 5.3 ± 0.7 times; for monkey B, the median number was 640.3 ± 0.4; presented an average of 4.3 ± 0.9 times). Tuning for orientation was measured using grayscale Gabor patches in a separate set of experiments, with six Gabor patches oriented at 0° to 150° in steps of 30° with 0° defined as the right horizontal meridian. Sites were defined as “orientation-selective” if they showed response modulation to the Gabor sets (*P* < 0.05, one-way ANOVA).

To quantify areal responses to photographs vs. artificial line stimuli (e.g., Gabor patches, bounded contour objects and spirals, *N* = 640 unique images across sets), first, we transformed each site’s responses to all images into z-score values (within that given experiment), in order to allow comparison across sites and experiments. Only sites whose RFs were located within 1° of the 3°-wide stimulus centers were examined. We computed each site’s maximum z-score value to all photographs and to all artificial line objects, and then measured the difference between both maximum values. These differences were then parsed per visual area and tested for statistical significance using a Kruskal-Wallis test (with areas as grouping variables) or Wilcoxon signed-rank test (for comparing V1/V2 and IT differences).

To determine if individual images contained dominant contour orientations, each image was run through MATLAB’s histogram-of-gradients (HOG) algorithm (*extractHOGFeatures.m)* to calculate the magnitude of unsigned gradient filters distributed across nine orientations *φ* = { *x* | 0° ≤ x ≤ 160°, *x* mod 20 = 0 } per image patch (block size = 9 pixels), denoted as | **∇**_**φ**_ | . At each patch, extracted strengths were used to create a local orientation dominance index as *I*_*local* =_ (max(**|∇**_**φ**_ | )-min(**|∇**_**φ**_ | )) / (max(**|∇**_**φ**_ | )+min(**|∇**_**φ**_ | )). Across images, most patches showed low orientation dominance because they either contained no strong edges or had meandering edges with no specific direction (e.g., a sand texture, or grid texture). Only patches with the strongest selectivity corresponded to striking features of the original image. Thus, to capture the orientation dominance of the image, we disregarded 75% of the patches and kept only the 25% most selective patches according to *I*_*local*_. We then computed the average (mean) oriented gradient magnitudes | **∇**_**φ**_ | across all retained patches. The final reported orientation dominance index per image was the orientation dominance index *I*_*global*_ computed for these mean oriented gradient magnitudes.

To semantically define the selectivity of individual sites, we analyzed the content of the images via Google Cloud Vision^19^, which returns multiple descriptive labels associated with each image. As a third-party resource trained on the world’s largest image dataset^19^, this label distribution provided a more comprehensive description of the images. For each experiment, a subset of images from the 754-sample datastore was used. First, we measured the experiment-specific label frequency (the null distributions in Fig. 2). Then for each site, we identified the images that elicited the highest firing rates and grouped those images’ associated labels (generally two images per site, corresponding to an average of 14.9 ± 0.5 labels per image). To determine if the frequency of any given label was disproportionately selected by sites in each array (IT, V4, V1/V2) relative to the null distributions, we conducted a bootstrap analysis where each null distribution was resampled 500 times using the same number of observed top labels across experiments and measured the frequency of each label within each bootstrap samples. Any given neuron-associated label was defined as disproportionately selected if its observed frequency fell outside the 99^th^ percentile of the null frequency distribution. On any given experiment, sites were only evaluated for selectivity if the stimulus center fell within 1.0° of the site’s RF center (RFs estimated in a different set of experiments, outlined above).

### Prototype synthesis

We performed 264 experiments in two animals (A and B, 127 and 137 experiments) using channels in V1/V2 (16 per monkey), V4 (16 per monkey), and PIT (32 for monkey A, 21 for B). Every site was tested at least once regardless of visual responsivity. In some analyses (for example, those in Figs. 1 and 2), 12 experiments conducted in monkey B were excluded because they involved channels with poor visual responsiveness, per the RF-mapping experiments; in analyses where shuffled prototypes were involved, those 12 experiments were used (as the failed attempted evolutions could be informative in defining compressibility values, for example). We defined the relative success of a prototype extraction experiment using two scores: (i) if the mean firing rate across the final 10 generations was higher than the mean firing rate to the first generation (with 95% CI of the difference between the final and initial firing rate not including zero) and (ii) if the mean firing rate to the synthetic images was larger than the mean firing rate to the reference images (which had been preselected due to their effectiveness in driving activity in that same array site; per the results, this was the more stringent test). More specifically, the evolution experiments relied on responses to single presentations of each image; this made the dataset noisier than those relying on average responses to multiple repetitions. For this reason, in the first analysis, (i) we designed a bootstrap test to minimize the effect of outliers. On each pass, we randomly sampled responses across the full experiment and sorted them by generation and by stimulus type (synthetic vs. reference). The site’s mean activity per generation was then computed separately for the synthetic and for the reference images, resulting in two vectors **r**_synthetic_ and **r**_reference_ (where each vector has *N*_*generation*_ elements). Each vector was smoothed with a moving average window of five generations; the response to the first generation was subtracted from the mean of the final 10 generations. We collected this difference across 500 iterations and computed the mean and standard error of this difference per experiment; the prototype extraction was considered successful if the 95% confidence interval of the bootstrap distribution did not include zero.

To measure the number of generations needed to reach a prototype, for each experiment, we convolved the mean firing rate per generation using a moving mean filter (window *N* = 5 generations). The amplitude of the curve was defined as the maximum value across generations minus the value at the first generation. The mid-point was defined as the generation where one-half of the amplitude was first reached.

### Semantic ensembles

To interpret the synthetic images across visual areas, we used ensembles of hidden units from AlexNet to score the likelihood of specific hypotheses about the enrichment of features such as straight contours, curvatures, color, faces, body parts, etc. For each hypothesis, hidden units were selected based on their responses to image sets with known properties. For example, to identify units sensitive to curved vs. straight contours, two image datastores were created, one containing curved contours (“banana Gabors”) and the other, straight contours; both datastores were propagated into the network and the activations of all units in each layer were recorded. AlexNet layer 6 was chosen because the generator used in this study was validated on it^20^, and AlexNet is more thoroughly vetted for predicting neuronal responses^21,65^. Because layer 6 follows all the convolutional layers, each unit’s responses involve all parts of the image, thus variation in feature position was irrelevant. The responses of each unit to both datasets were compared using a one-tailed Wilcoxon rank-sum test, and only units showing a statistically higher response to the first dataset (e.g., curved contours) were added to the semantic ensemble (*P* < 0.0001). Semantic ensembles comprised hundreds of units (Table 4). Hypothesis-defining image datastores comprised hundreds of images selected from various datasets including ImageNet^17^ and Caltech-256^66^. In one control, random data stores were created by sampling images randomly from the above datasets; in these cases, no units could be found that preferred one set over another, so random units were instead selected. After ensembles were identified, all prototypes were inputted into the network; the responses of each ensemble unit were z-scored across prototypes (but within units), and the mean response of each unit per areal prototypes was reported.

### Complexity metrics

To compute the mean complexity ratio for each image, we used MATLAB’s discrete cosine transform function (*dct2.m*). First, each color image was transformed to grayscale by retaining only the L* channel of the CIE 1976 L*a*b* color space. Next, pixel values were centered by subtracting the mean value. The image was then transformed into discrete cosine coefficients, which were then squared to yield the two-dimensional power-spectrum. To get the fraction of power contained in each component, the power of each component was divided by the sum of the entire power-spectrum. The components were then sorted in descending order by fractional power. The sum of the first *N* fractional power components yields the total fractional power contained in those *N* components. When they are sorted in descending order, this is the minimum *N* required to reach that fractional power. We designate nine fractional power levels ranging from 99.8% to 50%, per the formula *f* = 1–2 ^(−9 to −1)^) and find the minimum number of components, *N*, necessary to reach each fractional power level. To calculate the final complexity ratio, we average *N* across all nine fractional power levels and divide by the total number of components (which is equal to the number of pixels in the original image).

To compute the median number of parts that composed each prototype, mean-shift clustering was used. Each image was resized to dimensions of (100x100)-pixels, and then inputted into the custom function *Ms2.m*^34^ using the default bandwidth of 0.2; this particular function clusters pixels based on color and spatial relationships (proximity). It returns several estimated clusters along with the number of pixels associated with each cluster; we retained all clusters with more than 10 pixels.

### Random drift evolutions

To simulate synthesis experiments without neuronal guidance, computational simulations were set up as follows. Each simulation began by sampling an input vector obtained from an experiment and shuffling the position of its elements. This shuffled vector then served as a target for the evolution, leading to a different location in generator input space while still preserving the vector norm (and thus the distance traveled from the center of the generator input space). The first generation used the same set of input vectors as in the experiments. Each image was then scored based on the inverse of its distance to the target vector; Covariance Matrix Adaptation Evolutionary Strategy (CMA-ES) was used to identify new candidate vectors for the next generation. The evolution continued until the mean norm of a given generation reached the same norm as that of the target vector.

### Free viewing

*Behavior*. The head-fixed subjects were placed in front of a monitor and their eye positions were tracked with an ISCAN infrared gaze tracking device. To begin each trial, subjects held initial fixation on a 0.25°-diameter circle at the center of the screen for 100 ms, after which the fixation point was replaced by a full-color scene on a grey background. Scenes were presented one at a time at 15° × 15° for 1000 ms, during which time the subject was free to view anywhere within a 36° × 32° viewing window without triggering an error. Trials were aborted and restarted if their gaze left that window. After each trial, the subject received a fixed liquid reward. To control for the photographer’s bias and any confounds from the initial center fixation, the original color picture was randomly offset by 2° in any direction and embedded within a fixed gray frame of the same color as the monitor background.

The stimulus set comprised 5223 photographs from different sources, including 1) random pictures from the COCO dataset^67^, 2) natural scenes containing primates, and 3) pictures of familiar objects and personnel, taken around the animals’ home cage and our laboratory. Analysis of the photographs per Google Cloud Vision resulted in 53,000–56,000 labels (2913 to 2965 unique labels), with the most frequent labels comprising Vertebrate (1.5%), Mammal (1.5%), Vehicle (1.4%), Room (1.4%), Primate (1.3%), Old world monkey (1.0%) and Wildlife (1.3%).

*Analysis*. Calibrated eye position data from MonkeyLogic 2 was used for all analyses. Only successful trials were used (i.e., the monkey’s eye positions remained within the 36° × 32° window). Eye position data were analyzed using ClusterFix^68^, a MATLAB package that uses K-means clustering to parse out saccade times and fixation durations, based on velocity and acceleration. On each trial, this was used to find the location of the fixation following the first saccade after image onset: this image region was then cropped out using *imcrop.m* to yield a “scene patch.” As a control, we used the same eye position information and randomly selected an image from a different trial, then cropped out the region marked by the first acquired fixation to yield a *non-viewed* scene patch.

For the prototype-comparison analysis, viewed- and non-viewed fragments from a given animal were compared to its own prototypes as defined in other experiments. All fragments, along with prototypes and shuffled prototype surrogates were inputted into AlexNet, converting each into a layer fc6 activation vector (4096 × 1). Distance among vectors were computed using a correlation-based distance (*pdist2.m*).

### Global image statistics

The global image statistics used to measure the fractional changes reported in Supplementary Tables 1-3 and Supplementary Fig. 4 were defined and calculated as follows. All statistics were calculated on the luminance channel of the CIE 1976 L*a*b* color space of the patch and prototype images. The images to be analyzed were selected as follows: For monkeys, A and B when viewed and/or non-viewed patches were centered near the edge of the image they contained the gray background against which images were set. This was never the case for prototypes, so patches near enough to image edges to include the background were excluded. This left a minimum of 9749 patches in any patch data set (see Supplementary Table 1). It is necessary that the prototype data set have numerical parity with the patch data set. There were 127 and 119 evolution experiments for monkeys A and B. For each evolution experiment we selected 100 synthesized images, 20 arbitrary selections from each of the last 5 generations. Using this many images gives numerical parity with the patch data set, while selecting from the last 5 generations increases the diversity of the selected images while remaining at or near the algorithm’s point of convergence.

*Image entropy* ($S=-{\sum }_{i=0}^{255}p\left(i\right)\ast {{{{{\rm{ln}}}}}}\left(p\left(i\right)\right)$) and *energy* ($E={\sum }_{i=0}^{255}p{\left(i\right)}^{2}$) were calculated with 256 bins across the luminance depth of the image.

*Stationarity* was interpreted to mean that luminance variability has no positional dependence, and thus integration of luminance values in any direction would asymptotically approach ${\mu }_{{L}^{\ast }}\times (t+1)$, where $t$ is the number of integration steps (pixels). Hence, the stationarity statistic is the log-likelihood of assumptions we make about image stationarity. The first step was to resize the image to 256x256 pixels. Then the image was integrated (summed, *dx* = 1 pixel) in 5 directions, top- > bottom, bottom- > top, left- > right, right- > left, and center- > out. Each step of integration produces a vector of values ${\overrightarrow{{L}_{t,d}^{\ast }}}$ where $d$ denotes the direction of integration. For center-out integration this vector acquires more elements (dimensions) until there is one element for each pixel at the image perimeter. At each step of integration an empirical probability distribution is estimated for vector element values, noted as ${P}_{{{{{{\rm{t}}}}}}}\left({L}^{\ast }|{\overrightarrow{{L}_{t,d}^{\ast }}}\right)$. If the image followed a linear model, then this distribution would peak near the value ${\mu }_{{L}^{\ast }}\times (t+1)$ where ${\mu }_{{L}^{\ast }}$ is the mean luminance value of the entire image. Thus, the log of the probability of the expected peak given the empirical distribution was recorded for each integration step and noted as ${\Psi }_{t,d}={{{{{\rm{ln}}}}}}\left({P}_{{{{{{\rm{t}}}}}}}\left({\mu }_{{L}^{\ast }}\times \left(t+1\right)|{\overrightarrow{{L}_{t,d}^{\ast }}}\right)\right)$. When $t$ is small, this is essentially the log-likelihood of the mean luminance value along the edge, or near the center — otherwise it is the log-likelihood of our assumption that there are no positional dependencies on luminance variability for stationary images. The final stationarity value is the sum of all recorded values, ${\Sigma }_{t,d}{\Psi }_{t,d}$, and this is validated through visual inspection of results.

In Tamura’s texture statistics^42^, the only free parameter is the choice of regularization factor for regularity, which was set at 3000. Prior to computing Tamura texture statistics on patches, the largest central most portion was cropped and resized to 256 × 256 (e.g., a 192 × 256 image would lose the topmost and bottom most 32 pixels and be rescaled to 256x256). Generator images were already 256 × 256.

In Supplementary Table 3 we used signed and unsigned differences. To capture unsigned differences (in Supplementary Table 2) we use ${d}_{j,i,k}=\left({s}_{j,k}-{b}_{j,i}\right)$ where ${s}_{j,k}$ is the value of the ${j}^{{th}}$ statistic for the ${k}^{{th}}$ surrogate image (either a shuffled prototype or a not-viewed patch) and ${s}_{j,i}$ is the value of the ${j}^{{th}}$ statistic for the ${i}^{{th}}$ baseline image (either a true prototype or a viewed patch). We only compared prototypes to prototypes and patches to patches. For Supplementary Fig. 4 we also normalize the pairwise difference for plotting by dividing pairwise differences by the global mean across all data sets for the ${j}^{{th}}$ statistic. For computing absolute (unsigned) fractional differences in Supplementary Table 3 we also took the absolute value and divided by the baseline: ${u}_{j,i,k}=\left|\left({s}_{j,k}-{b}_{j,i}\right)/{b}_{j,i}\right|$. We drew our conclusions about similarity from the absolute fractional differences because it accounted for both changes in both the location and dispersion of the image statistic distributions.

### COCO-stuff semantic label statistics

Here we describe our approach to measuring semantic content in different image sets (e.g., prototypes, freely viewed patches). In order to compare the content of these images, we leveraged the open-ended labeling abilities of deep neural networks. However, first it was necessary to restrict the set of labels to ensure overlap between the labels applied to disparate types of images (e.g., patches and prototypes). With Google Cloud Vision, few labels were found frequently enough in each class of images to make useful comparisons. Hence, we used a ResNet architecture^69,70^ trained on the COCO-Stuff semantic-segmentation labeled dataset^43^. This is one of the datasets from which free-viewing stimuli were drawn. COCO-Stuff augments the COCO dataset with additional annotations for non-object like categories (e.g., backgrounds like grass or sky). It has 182 labels with four hierarchical groupings. The fourth level is all 182 base labels, the third is 27 semantic categories. These labels and hierarchies are shown in Supplementary Table 4. We focused our analysis on the 27 semantic categories, but only those which were most abundantly (and therefore confidently) identified. We had eight data sets: viewed patches, not-viewed patches, true prototypes, shuffled prototypes each repeated per monkey. If a category’s occurrence frequency was greater than two standard deviations above zero in one or more of the eight datasets, it was included in our analysis (Fig. 4f, g), and we performed a hypothesis test. Only 7/27 categories met this criterion. The hypothesis test was a *Χ*^2^ proportion test between the presence or absence of a category in viewed vs non-viewed patch or true vs. shuffled prototypes, the significance threshold is set at 1.8519 × 10^−3^ (Bonferroni adjustment for *N* = 27).

The ResNet was designed to apply one label (or no label) to each pixel on the image using a conditional random field. We modified the architecture to apply multiple labels to each pixel to reduce the impact of mis-labeling, and to increase the likelihood that different images would have one or more labels in common. The process for assigning multiple labels to each pixel was as follows: initially, the ResNet produced a probability map for each label for all pixels and then a conditional random field assigned one label to each pixel given the probability of neighboring pixels. The ResNet was run only once. Each label that occupied an area greater than 1/9^th^ of the total image area was identified as a “dominant label”. The probability of dominant labels was set to the minimum probability value of the ResNet output. The probabilities were re-normalized and again inputted to the conditional random field. This produced a new set of labels and the process starting with identifying “dominant labels” was repeated nine more times. Each of the ten one-label maps given as outputs by the conditional random field was retained. Finally, all labels which occupied more than 1/9^th^ of the total image area in any one-label map were recorded as the main set of labels for the image.

The Python scripts managing the ResNet architecture and process were interfaced with the main MATLAB analysis scripts using the built-in interfacing capabilities of MATLAB. Images were processed with 30 parallel computational threads. After several days, 1,528 randomly selected viewed patch images from monkey A were processed (see Global image statistics above for sample curation details). The decision was made to cut off analysis after 1000 images from each data set were processed. For patches, images were processed in random order for each group (viewed/not-viewed, monkey A/monkey B). For prototypes, each experiment was processed two images at a time. We selected one image from the five last generations of evolving prototypes and its shuffled counterpart. We then moved on to the next experiment, looping through again until all experiments had seven or more prototypes (fourteen or more images) processed. Thus, the frequency statistics account for 948 or more images from each data set.

### Statistics and reproducibility

Each major result was based on over 120 experimental sessions carried out using the same design and equipment type, in two separate animals, following standard practice in non-human primate electrophysiology.

## Supplementary information

Supplementary Information

Supplementary Information

## Data Availability

All correspondence and material requests should be addressed to the corresponding author (C.R.P). Data generated in this study have been deposited in an Open-Science Framework repository^71^ at the URL https://osf.io/z6gv2/. We may share more data in the future, so a timestamped version of the repository at the time of publication is also found at https://osf.io/z6gv2/. Data can be obtained by downloading a zip file and can be loaded using MATLAB. Raw spike data and peristimulus histograms are undergoing analyses and are not yet available. Processed data to illustrate each analysis and figure are available at Github (https://github.com/PonceLab/as-simple-as-possible). This Github repository is linked to the aforementioned Open-Science Framework repository. We have shared the latent vectors obtained during image synthesis experiments and responses to the images evoked by those latent vectors, along with sufficient metadata to reproduce most of the results in the publication. Data provided in the OSF repository can be used to replicate Fig. 1e, f and Fig. 2a, b. In conjunction with code from the GitHub repository, the data can also be used to recreate Fig. 2d, e and Fig. 3c, e. Results featured in Fig. 4 are based on eye movements applied to images for which we hold no copyright and include pictures depicting identifiable humans, who have not consented for the distribution of their pictures in the context of this work. Thus we have not released the gaze behavior data, nor the baseline reference images due to concerns about privacy and distribution of possibly copyrighted material. However, all of these data are available upon request for research purposes, with guarantees of confidentiality, by contacting the corresponding author. Source data for (supplementary) tables are very large and will be available upon request by contacting the corresponding author. Data used for stimuli and analyses include ImageNet (https://image-net.org/) and COCO-Stuff (https://github.com/nightrome/cocostuff). Source data are provided with this paper.

## Source data

Source Data

## Code availability

Code and links to useful repositories that can be used to reproduce most of the results and figures (see Data Availability statement) are deposited in a Github repository (https://github.com/PonceLab/as-simple-as-possible). The link to the timestamped Zenodo repository is 10.5281/zenodo.5228069^72^.
