# Supplementary material for: Visual prototypes in the ventral stream are attuned to complexity and gaze behavior
Source: Nat Commun. 2021 Nov 18;12:6723. doi: 10.1038/s41467-021-27027-8 (PMC8602238; doi:10.1038/s41467-021-27027-8)
Supplement: Supplementary file 1 — Supplementary Information [file 41467_2021_27027_MOESM1_ESM.pdf]

## SUPPLEMENTARY INFORMATION for

# Visual prototypes in the ventral stream are attuned to complexity and gaze behavior

Rose O., Johnson J.K., Wang B., and Ponce C.R.

**Supplementary Figure 1.**

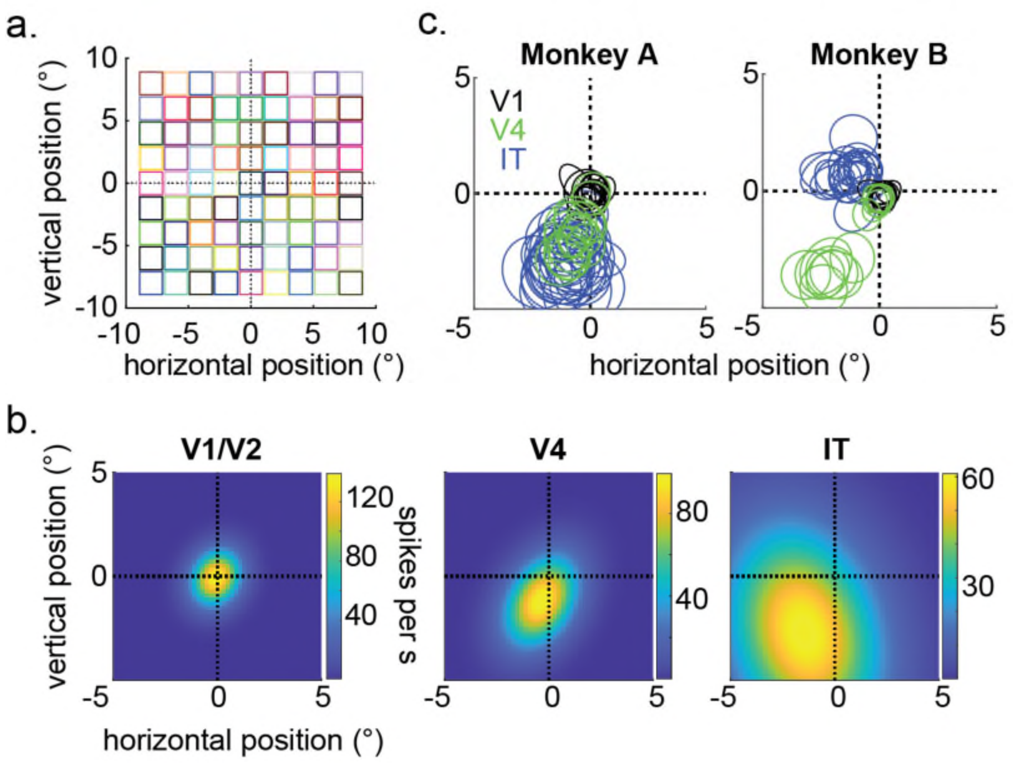


**Receptive field center mapping. a.** Each square in the grid shows one possible location of an 2°-wide image, presented for 100 ms, while the monkey fixated its gaze at (0,0). The image was presented at one single location at any given time. Colors used to illustrate different positions (a map coloring). **b.** Gaussian functions fit to observed spike rate data, plotted as a function of retinotopic space, for three different multiunit sites. **c.** Outlines show locations of thresholded Gaussian fits (i.e. contours) per site (75% of peak activity), across visual areas (indicated with color per legend, V1/V2 = black, V4 = green, IT = blue). Source data are provided as a Source Data file.

**Supplementary Figure 2**

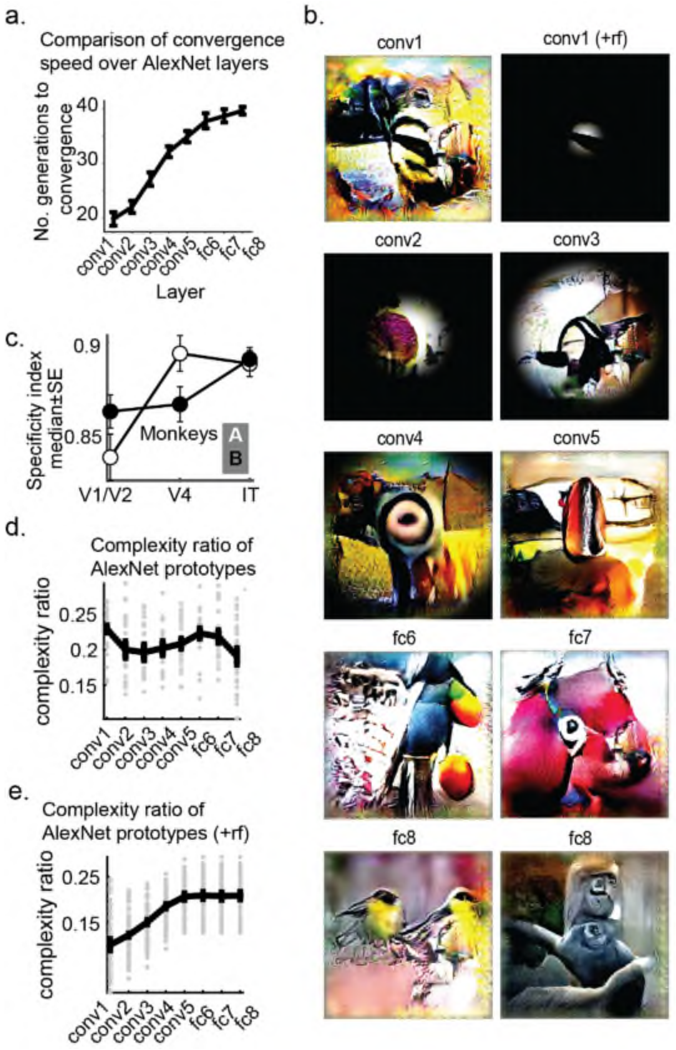


**Units in deeper hierarchical levels take longer to construct their stimulus prototypes.** To determine if hierarchical depth is a predictor of convergence speed (i.e. the number of generations needed to extract a local prototype), we performed experiments using units in the convolutional neural network AlexNet. The same procedure as described in the main text was used to extract prototypes for these model neurons/hidden units, with each experiment running for 100 iterations. For convolutional layers, we chose the units responding to the center of the feature map, just as we placed the synthetic images at the receptive field center of neurons in the biology experiments. First, we conducted experiments where we used a fixed stimulus size (224 pixels) for all layers. Thus, mimicking the neurophysiology experiments, where the stimulus extended beyond the classical RF. Each successive AlexNet layer accumulates more features which must be present across the visual field in order to achieve maximum activation. Thus, hierarchical depth is associated with greater specificity in the types of images that will achieve maximum activation. It is harder to solve an optimization problem that is more specific in the requirements that must be satisfied, thus it is expected to take longer to optimize stimuli for later layers. We validated this model-based reasoning to show that higher cortical regions also show greater specificity in the prototypes they require. Thereby explaining the greater time to convergence.

**a.** Number of generations needed to reach 50% of the maximal activation (specifically the mean activation per generation) as a function of layer. Units across experiments were pooled. We found a clear trend that units deeper in the hierarchy require more time to converge. Similar results were found when we repeated the experiments resizing the image to match the precise “receptive field” for the unit (i.e. from 11x11 pixels for

Conv1 to 224 pixels for FC8). Data shown are mean number of generations ± standard error of the mean (error bars). Source data are provided as a Source Data file.

**b.** Example prototypes from randomly chosen channels from Conv1 to FC8 (in convolutional layers, the hidden unit RF is at the center). The black mask shows each unit's receptive field diameter ("rf").

**c.** In order to test the idea that IT prototypes were more selective or specific than V1 prototypes (thus posing more difficult optimization problems), we developed an index defined as the mean of pairwise best-case image correlations, after controlling for possible translation, scale, and phase invariance. The more specificity in the location and pixel-level details of features in the image the higher the best-case correlation. This *specificity index* is computed using all images presented in the last generation of the evolution experiment. The "best-case image correlation" is calculated as follows: for each image in each pair, we converted to an opponent color space (CIELAB) and compute all combinations of translation and rescaling then downsampled to 32 x 32 pixels. This produced a large set of transformed images for each image in the pair. Next, we tested phase invariance by subtracting a 3x3 median filtered version of each transformed images and computing the absolute value (full wave rectification). This doubled the number of transformed images (rectified and not rectified) for each image in the pair to yield K transformed versions of each image in the pair. We then computed the pixel-wise Pearson correlation coefficient $K^2$ times to compare each transformed version of one image in the pair to each transformed version of the other image. The "best-case image correlation" was the max of this set of these $K^2$ correlations. Each image was originally 256 x 256 pixels and the maximum translation was 32 pixels, making translated images 225 x 225. We tested the translations [1,11,22,32] pixels in the vertical and horizontal directions. We also included the original 256 x 256 image to make a total of 17 translations. We cropped to the central [100,93,87,80]% of each translated image, producing 68 translated and rescaled images. Rescaling and translation and phase invariance produced 136 transformed versions of each image. This allowed us to test the extent to which evolutions produced variations on the same image, and how much variation was present (while acknowledging that neurons may not be able to detect small changes in translation, scale and apparent angle of illumination). We found the median specificity index values for V1 were 0.84±0.01 and 0.86±0.01 (monkeys A and B) while for IT, it was 0.89±0.01($P$ = 3 x $10^{-4}$ and 8 x $10^{-3}$, Wilcoxon rank sum test, two-sided, $r_{sdf}$ -0.3 – -0.5). Data shown are median ± standard error (error bars; n = number of hidden unit prototypes, conv1, 30; conv2, 29; conv3, 29; conv4, 27; conv5, 27; fc6, 18; fc7, 25; fc8, 33). Monkey A = white; B = black. Source data are provided as a Source Data file.

**d.** In a different experiment, we also measured the compression ratio for AlexNet representations. To do this, we randomly selected 218 units from eight layers (conv1–5, fc6, fc7, fc8, 27.3±1.7 per layer), then used their activations to guide evolutions as in the biology experiments, using the same starting generator input codes, generator and search algorithm (CMA-ES), for a total of 100 generations (as in **a.**). We then computed the complexity ratio over the whole image. We found that most prototypes had a median complexity ratio of 0.209±0.008 (across layers; the median value varied across layers ($P$ = 9.4x$10^{-3}$, $\chi^2$= 18.7, Kruskal-Wallis test, two-sided). Data shown are median ± standard error (error bars). Source data are provided as a Source Data file.

**e.** To measure the effect of receptive field (RF) size, we also measured complexity ratio when the evolved images were masked with an aperture corresponding to the known size of the layer RF (in circular form to simulate a more biological RF). To do this, we randomly sampled 100 evolved images and masked them each with differently sized RF masks. We found that complexity ratio values scaled as a function of the displayed image. Data shown are median ± standard error (error bars, same *n* as **d**). Source data are provided as a Source Data file.

**Supplementary Figure 3.**

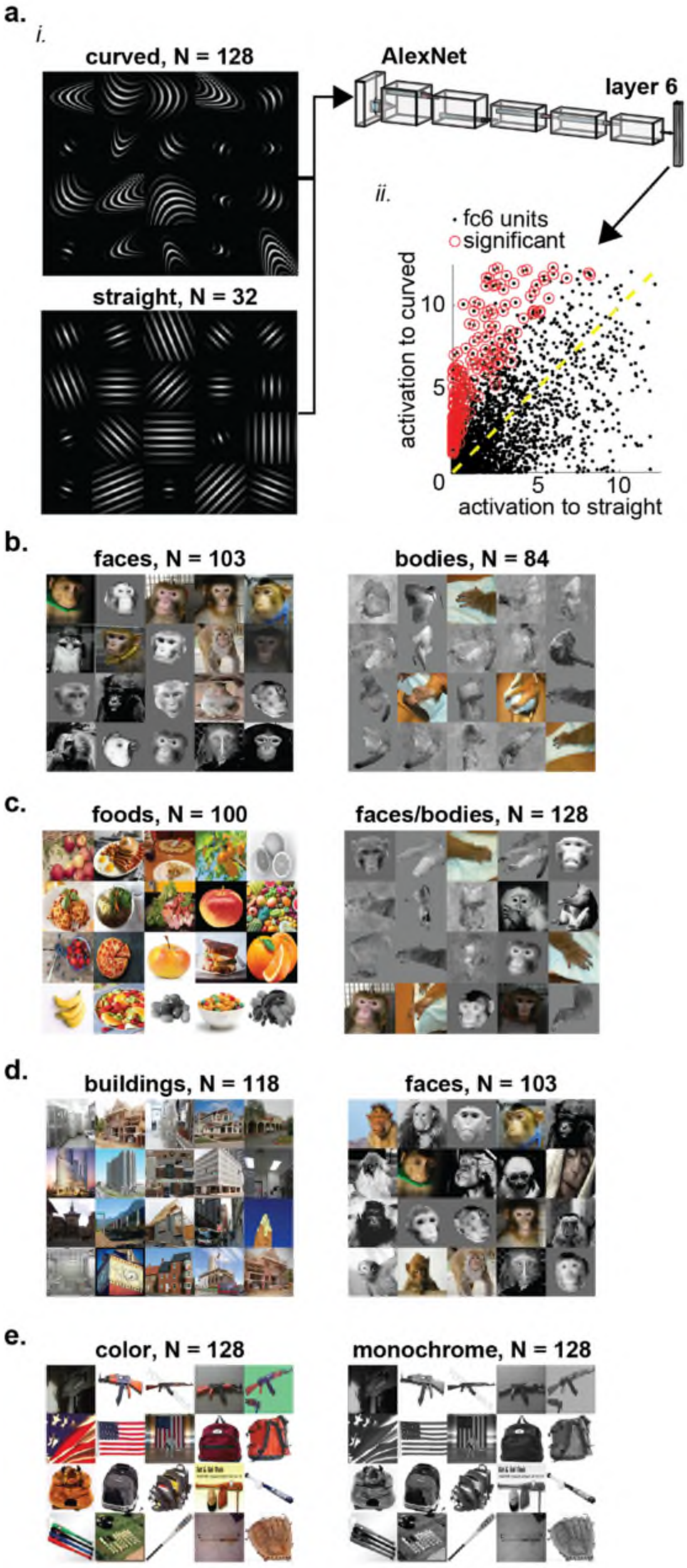


**Semantic ensembles. a.** (*i*) In this approach, first, two sets of images are selected in order to highlight a visual feature of interest, such as curvature. The image sets are propagated into AlexNet; (*ii*) units in layer ReLu6 that respond more strongly to the first set ("curved") than to the second set ("straight") are identified as the test ensemble (in each test, each tested unit must lead to a *P*-value < 0.0001 per a Wilcoxon rank sum test, one-sided, no correction for multiple comparisons). Afterwards, the activity of the unit ensemble to the independently obtained neuronal prototypes are then quantified as a function of cortical area (n = fc6 hidden units, 4096). Each dot shows the mean response of the unit to curved- or straight Gabor grating images, and red shows the statistically significant units. Source data are provided as a Source Data file. **b-e.** Image sets used to test other semantic hypotheses such as *faces vs. bodies*, *foods vs. faces/bodies*, *buildings vs. faces* and *color vs. grayscale*.

**Supplementary Figure 4.**

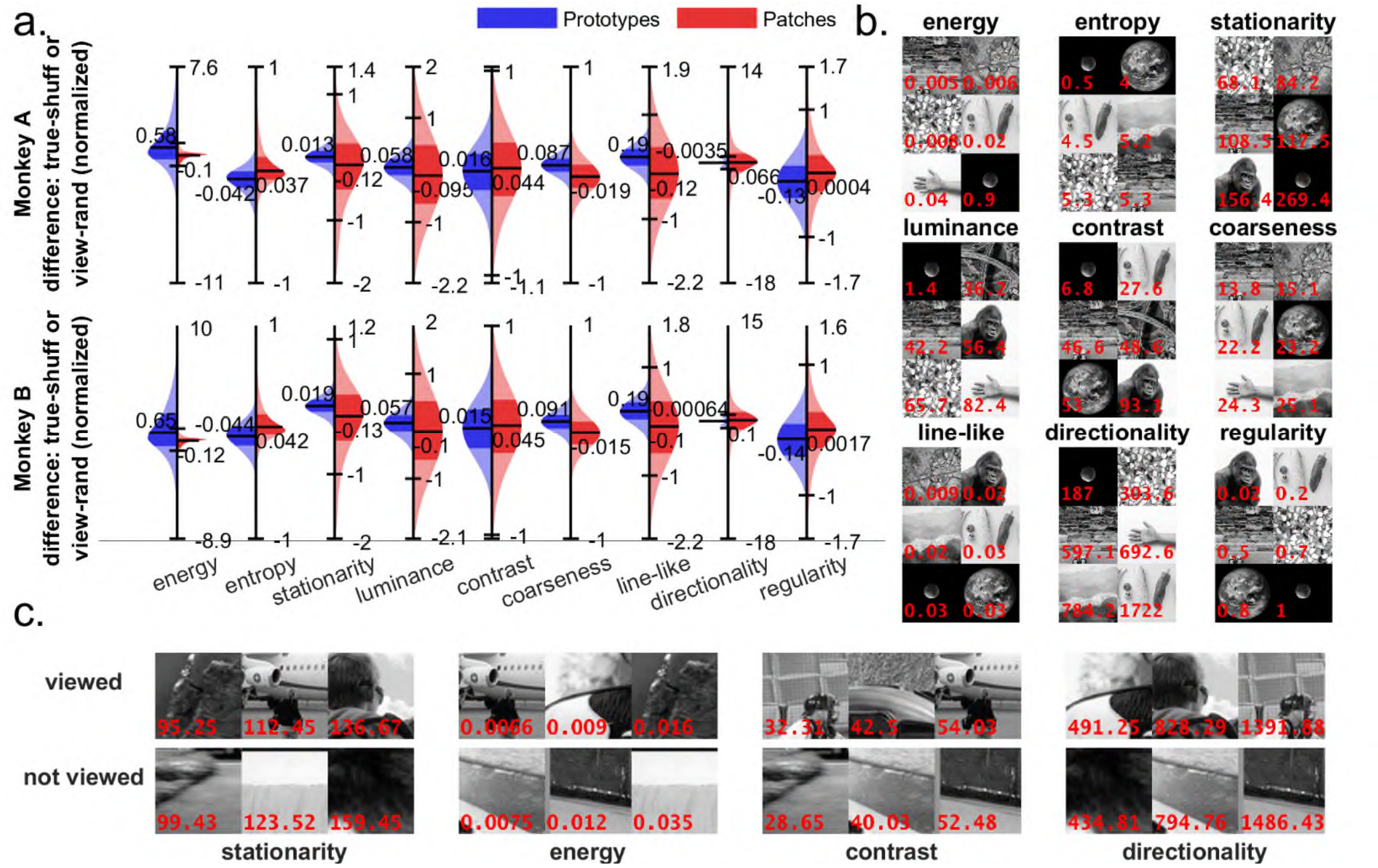


**Prototype shuffling perturbs low-level image properties less than natural scene patch randomization.**

**a.** Violin plots comparing the distribution of normalized differences (subtracting the statistic value) between each shuffled prototype from each observed prototype (blue) to the differences between any not-viewed patch from any viewed patch (red). For each violin plot, the median value of the statistic (for all images, across all monkeys) was divided out to normalize the plots and make them unitless. Nine common image statistics were chosen as a basis for making comparisons. Intensely colored regions show the 25th to 75th percentile of analyzed data, lighter colorization shows data to the 1st and 99th percentile of analyzed data, gray is beyond that (mostly cut off for plotting). There are a minimum of 9,749 images included, yielding a minimum of 104,645,766 pairwise comparisons in these distributions. Tailed Wilcoxon ranksum tests yielded *P*-values of zero to single precision, showing that patches have larger differences for all comparisons. Effect sizes are in **Supplementary Table 2**. Source data are provided as a Source Data file.

**b.** Examples of natural images and the values of the image statistics, ranging from least (upper left) to greatest (lower right) in each subplot. Statistics are performed only on the luminance channel of the CIE 1976 L*a*b* color space so only that channel is shown. The value is annotated on the plot.

**c.** The CIE 1976 L*a*b* luminance channel of patch images (viewed patches on top row, not-viewed on bottom) selected to illustrate the range of values for the stationarity, energy, contrast, and directionality statistics. The three images for each statistic and type of patch are within 0.5% of the 25th 50th and 75th percentile from left to right. Images are repeated by designing the plotting algorithm to select the fewest number of images possible to make the plot across all three statistics and percentiles.

# Supplementary Tables

| Supplementary Table 1. Global image statistic shifting effects: medians patches and prototypes (± half interquartile range) | | | | | | | | | | |
|---|---|---|---|---|---|---|---|---|---|---|
| Monkey A | N – see methods | energy median | entropy median | stationarity median | luminance median | contrast median | coarseness median | line-like median | directionality median | regularity median |
| viewed patches | 9749 | 0.0091±0.0045 | 5.01±0.26 | 113±20.5 | 123±26.6 | 42.6±10.7 | 23±1.46 | 0.054±0.013 | 835±449 | 0.22±0.077 |
| not-viewed patches | 10734 | 0.012±0.014 | 4.82±0.48 | 124±29.6 | 129±28.5 | 40±11.9 | 23.4±1.66 | 0.059±0.015 | 790±516 | 0.22±0.081 |
| Untailed ranksum test with p-value (P) and simple difference formula effect size (R): P,R | | $1.2x10^{-158}$, -0.22 | $1.7x10^{-182}$, 0.23 | $4.5x10^{-109}$, -0.18 | $3.3x10^{-29}$, -0.091 | $2.6x10^{-29}$, 0.091 | $1.2x10^{-31}$, -0.095 | $7.4x10^{-56}$, -0.13 | 0.00027, 0.029 | 0.92, 0.00087 |
| true prototypes | 12700 | 0.045±0.026 | 4.69±0.32 | 87.7±6.85 | 58.7±8.51 | 73.8±8.5 | 20.1±0.97 | 0.037±0.0046 | 314±124 | 0.49±0.076 |
| shuffled prototypes | 12700 | 0.031±0.015 | 4.9±0.22 | 86.6±5.71 | 55±7.44 | 72.6±6.74 | 18.3±0.86 | 0.029±0.0043 | 310±139 | 0.54±0.073 |
| Untailed ranksum test. | | 0, 0.29 | 0, -0.3 | $2.9x10^{-24}$, 0.074 | $5.2x10^{-162}$, 0.2 | $6.5x10^{-12}$, 0.05 | 0, 0.64 | 0, 0.58 | 0.11, -0.012 | $1.1x10^{-315}$, -0.28 |
| | | | | | | | | | | |
| Monkey B | N – see methods | energy median | entropy median | stationarity median | luminance median | contrast median | coarseness median | line-like median | directionality median | regularity median |
| viewed patches | 13775 | 0.0082±0.0033 | 5.06±0.22 | 110±18.5 | 121±26 | 42.6±10.3 | 23.1±1.48 | 0.055±0.012 | 837±436 | 0.22±0.074 |
| not-viewed patches | 10564 | 0.011±0.01 | 4.85±0.43 | 122±27.9 | 129±29.4 | 39.9±11.9 | 23.4±1.63 | 0.059±0.014 | 787±484 | 0.21±0.078 |
| Untailed ranksum test. | | $4.3x10^{-315}$, -0.28 | 0, 0.29 | $7.1x10^{-175}$, -0.21 | $2.4x10^{-39}$, -0.098 | $2.3x10^{-36}$, 0.094 | $4.1x10^{-24}$, -0.076 | $2.6x10^{-50}$, -0.11 | $4.5x10^{-10}$, 0.047 | 0.63, 0.0037 |
| true prototypes | 11900 | 0.052±0.03 | 4.61±0.35 | 88.7±6.82 | 59.4±7.57 | 74.7±9.15 | 20.1±0.99 | 0.037±0.0045 | 311±120 | 0.48±0.07 |
| shuffled prototypes | 11900 | 0.035±0.019 | 4.84±0.26 | 87±5.89 | 55.1±7.51 | 73.7±7.37 | 18.2±0.89 | 0.029±0.0042 | 309±139 | 0.54±0.073 |
| Untailed ranksum test. | | $1.5x10^{-284}$, 0.27 | $1.4x10^{-306}$, -0.28 | $7.5x10^{-48}$, 0.11 | $3.3x10^{-154}$, 0.2 | $1.6x10^{-8}$, 0.042 | 0, 0.67 | 0, 0.6 | 0.77, -0.0022 | 0, -0.31 |
| | | | | | | | | | | |
| global | 94022 | 0.022 | 4.86 | 94.2 | 70.1 | 61.9 | 20.7 | 0.039 | 490 | 0.42 |

| Supplementary Table 2. Global image statistic relative shifting effect: pairwise difference with surrogates in patches and prototypes | | | | | | | | | |
|---|---|---|---|---|---|---|---|---|---|
| Monkey A | energy | entropy | stationarity | luminance | contrast | coarseness | line-like | directionality | regularity |
| change between true and shuffled prototypes median ± half IQR | 0.013±0.028 | -0.2±0.37 | 1.18±8.7 | 4.07±11.1 | 1.01±11 | 1.79±1.3 | 0.0075±0.0063 | -1.73±139 | -0.055±0.11 |
| change between viewed and not-viewed patches | -0.0022±0.012 | 0.18±0.51 | -11.1±35.1 | -6.64±39.4 | 2.73±16.3 | -0.39±2.22 | -0.0046±0.02 | 32.5±673 | 0.00017±0.12 |
| Untailed ranksum test for patch shift relative to prototype shift with | 0, -0.36 | 0, 0.38 | 0, -0.2 | 0, -0.14 | 0, 0.055 | 0, -0.42 | 0, -0.31 | 0, 0.028 | 0, 0.17 |

| | | | | | | | | | |
|---|---|---|---|---|---|---|---|---|---|
| simple difference formula effect size "R" and P-value “P”. R<0 implies patch disparity was less positive. Ordered P, R | | | | | | | | | |
| | | | | | | | | | |
| Monkey B | energy | entropy | stationarity | luminance | contrast | coarseness | line-like | directionality | regularity |
| change between true and shuffled prototypes | 0.64±0.26 | -0.21±0.42 | 1.76±8.77 | 4±10.7 | 0.91±11.5 | 1.88±1.31 | 0.0077±0.0062 | -0.31±134 | -0.059±0.1 |
| change between viewed and not-viewed patches | -0.0026±0.0098 | 0.2±0.46 | -12.2±32.9 | -7.18±39.4 | 2.77±15.9 | -0.31±2.21 | -0.0039±0.019 | 49.7±642 | 0.00071±0.11 |
| P, R for ranksum test for patch shift relative to prototype shift. | 0, -0.38 | 0, 0.41 | 0, -0.24 | 0, -0.14 | 0, 0.051 | 0, -0.43 | 0, -0.31 | 0, 0.046 | 0, 0.19 |

| Supplementary Table 3. Global image statistic combined spreading and shifting: absolute fractional pairwise difference with surrogates in patches and prototypes | | | | | | | | | |
|---|---|---|---|---|---|---|---|---|---|
| Monkey A | energy | entropy | stationarity | luminance | contrast | coarseness | line-like | directionality | regularity |
| absolute fractional change between true and shuffled prototypes<br>median ± half IQR | 0.62±0.25 | 0.081±0.06 | 0.099±0.061 | 0.2±0.11 | 0.15±0.094 | 0.097±0.051 | 0.24±0.12 | 0.35±0.29 | 0.22±0.15 |
| absolute fractional change between viewed and not-viewed patches | 0.71±1.07 | 0.099±0.095 | 0.29±0.2 | 0.32±0.21 | 0.39±0.23 | 0.098±0.064 | 0.36±0.24 | 0.73±0.48 | 0.49±0.34 |
| Untailed ranksum test for patches showing greater disparity than prototypes, with simple difference formula effect size "R" and P-value “P”. R<0 implies viewed/not-viewed patches showed less disparity and vice versa. Ordered as P, R. | 0, 0.15 | 0, 0.12 | 0, 0.58 | 0, 0.31 | 0, 0.5 | 0, 0.044 | 0, 0.3 | 0, 0.38 | 0, 0.41 |
| | | | | | | | | | |
| Monkey B | energy | entropy | stationarity | luminance | contrast | coarseness | line-like | directionality | regularity |
| absolute fractional change between true and shuffled prototypes | 0.64±0.26 | 0.094±0.066 | 0.1±0.061 | 0.19±0.11 | 0.15±0.095 | 0.1±0.053 | 0.24±0.12 | 0.34±0.29 | 0.22±0.15 |
| absolute fractional change between viewed and not-viewed patches | 0.65±0.97 | 0.087±0.08 | 0.28±0.19 | 0.32±0.21 | 0.38±0.22 | 0.096±0.063 | 0.34±0.22 | 0.71±0.4 | 0.48±0.32 |

| | | | | | | | | | |
|---|---|---|---|---|---|---|---|---|---|
| P, R for untailed ranksum test for patches showing greater disparity than prototypes | 0, 0.085 | 0, -0.0011 | 0, 0.57 | 0, 0.32 | 0, 0.5 | 0, 0.015 | 0, 0.27 | 0, 0.37 | 0, 0.42 |

| Supplementary Table 4. Sublabels for each of the 27 semantic categories, with higher grouping levels noted. | | |
|---|---|---|
| accessory<outdoor<things:<br>hat, backpack, umbrella, shoe, eye glasses, handbag, tie, suitcase | furniture<indoor<stuff:<br>cabinet, counter, cupboard, desk-stuff, door-stuff, furniture-other, light, mirror-stuff, shelf, stairs, table | sky<outdoor<stuff:<br>clouds, sky-other |
| animal<outdoor<things:<br>bird, cat, dog, horse, sheep, cow, elephant, bear, zebra, giraffe | furniture<indoor<things:<br>chair, couch, potted plant, bed, mirror, dining table, window, desk, toilet, door | solid<outdoor<stuff:<br>hill, mountain, rock, solid-other, stone, wood |
| appliance<indoor<things:<br>microwave, oven, toaster, sink, refrigerator, blender | ground<outdoor<stuff:<br>dirt, gravel, ground-other, mud, pavement, platform, playingfield, railroad, road, sand, snow | sports<outdoor<things:<br>frisbee, skis, snowboard, sports ball, kite, baseball bat, baseball glove, skateboard, surfboard, tennis racket |
| building<outdoor<stuff:<br>bridge, building-other, house, roof, skyscraper, tent | indoor<indoor<things:<br>book, clock, vase, scissors, teddy bear, hair drier, toothbrush, hair brush | structural<outdoor<stuff:<br>cage, fence, net, railing, structural-other |
| ceiling<indoor<stuff:<br>ceiling-other, ceiling-tile | kitchen<indoor<things:<br>bottle, plate, wine glass, cup, fork, knife, spoon, bowl | textile<indoor<stuff:<br>banner, blanket, cloth, clothes, curtain, mat, napkin, pillow, rug, textile-other, towel |
| electronic<indoor<things:<br>tv, laptop, mouse, remote, keyboard, cell phone | outdoor<outdoor<things:<br>traffic light, fire hydrant, street sign, stop sign, parking meter, bench | vehicle<outdoor<things:<br>bicycle, car, motorcycle, airplane, bus, train, truck, boat |
| floor<indoor<stuff:<br>carpet, floor-marble, floor-other, floor-stone, floor-tile, floor-wood | person<outdoor<things:<br>person | wall<indoor<stuff:<br>wall-brick, wall-concrete, wall-other, wall-panel, wall-stone, wall-tile, wall-wood |
| food<indoor<stuff:<br>food-other, fruit, salad, vegetable | plant<outdoor<stuff:<br>branch, bush, flower, grass, leaves, moss, plant-other, straw, tree | water<outdoor<stuff:<br>fog, river, sea, water-other, waterdrops |
| food<indoor<things:<br>banana, apple, sandwich, orange, broccoli, carrot, hot dog, pizza, donut, cake | Raw material<indoor<stuff:<br>cardboard, metal, paper, plastic | window<indoor<stuff:<br>window-blind, window-other |

| Supplementary Table 5. Occurrence frequency for COCO-stuff semantic categories | | | |
|---|---|---|---|
| Monkey A | N – see methods | Frequency standard deviation | Selected categories and frequencies |
| viewed patches | 1528 | 0.027 | animal: 0.074, electronic: 0.015, furniture: 0.065, ground: 0.082, plant: 0.091, textile: 0.054, wall: 0.11 |
| not-viewed patches | 1074 | 0.031 | animal: 0.059, electronic: 0.018, furniture: 0.076, ground: 0.11, plant: 0.091, textile: 0.046, wall: 0.12 |
| true prototypes | 1111 | 0.044 | animal: 0.2, electronic: 0.1, furniture: 0.021, ground: 0.048, plant: 0.07, textile: 0.088, wall: 0.096 |
| shuffled prototypes | 1111 | 0.044 | animal: 0.17, electronic: 0.11, furniture: 0.013, ground: 0.041, plant: 0.099, textile: 0.096, wall: 0.11 |
| | | | |
| Monkey B | N – see methods | Frequency standard deviation | Selected categories and frequencies |
| viewed patches | 1072 | 0.027 | animal: 0.09, electronic: 0.014, furniture: 0.056, ground: 0.083, plant: 0.092, textile: 0.058, wall: 0.11 |
| not-viewed patches | 1010 | 0.031 | animal: 0.053, electronic: 0.017, furniture: 0.073, ground: 0.11, plant: 0.096, textile: 0.045, wall: 0.12 |
| true prototypes | 949 | 0.045 | animal: 0.2, electronic: 0.1, furniture: 0.019, ground: 0.047, plant: 0.067, textile: 0.085, wall: 0.099 |
| shuffled prototypes | 948 | 0.044 | animal: 0.17, electronic: 0.1, furniture: 0.013, ground: 0.04, plant: 0.11, textile: 0.09, wall: 0.11 |

| Supplementary Table 6. Chi square statistics for seven categories | | | | | | | |
|---|---|---|---|---|---|---|---|
| Monkey A | animal | textile | electronic | furniture | ground | wall | plant |
| Chi-square proportion test for the relative abundances in viewed vs not-viewed patches. P-value "P", and Chi-squared statistic "$X^2$", in order: P, $X^2$ | $7.7x10^{-6}$, 20 | 0.039, 4.3 | 0.0023, 9.3 | $4.3x10^{-9}$, 35 | $2.3x10^{-12}$, 49 | 0.0048, 7.9 | 0.36, 0.83 |
| X-square proportion test for the relative abundances in true vs shuffled prototypes. | $7.4x10^{-12}$, 47 | 0.36, 0.85 | 0.00078, 11 | $8.9x10^{-9}$, 33 | 0.86, 0.029 | $6.8x10^{-11}$, 43 | $1.1x10^{-9}$, 37 |
| | | | | | | | |
| Monkey B | animal | textile | electronic | furniture | ground | wall | plant |
| Chi-square proportion test for the relative abundances in viewed vs not-viewed patches. | $1.4x10^{-12}$, 50 | 0.00011, 15 | 0.1, 2.7 | $1.2x10^{-8}$, 32 | $4.4x10^{-11}$, 43 | $4.1x10^{-6}$, 21 | 0.32, 1 |
| Chi-square proportion test for the relative abundances in true vs shuffled prototypes. | $3.2x10^{-5}$, 17 | 0.26, 1.3 | 0.43, 0.62 | $5.5x10^{-6}$, 21 | 0.47, 0.52 | $1.9x10^{-6}$, 23 | $1.3x10^{-17}$, 73 |
