# Supplementary material for: Visual prototypes in the ventral stream are attuned to complexity and gaze behavior
Source: Nat Commun. 2021 Nov 18;12:6723. doi: 10.1038/s41467-021-27027-8 (PMC8602238; doi:10.1038/s41467-021-27027-8)
Supplement: Supplementary file 2 — Supplementary Information [file 41467_2021_27027_MOESM2_ESM.pdf]

nature portfolio

Corresponding author(s): Ponce, Carlos R.

Last updated by author(s): Aug 20, 2021

Reporting Summary

Nature Portfolio wishes to improve the reproducibility of the work that we publish. This form provides structure for consistency and transparency in reporting. For further information on Nature Portfolio policies, see our Editorial Policies and the Editorial Policy Checklist.

Statistics

For all statistical analyses, confirm that the following items are present in the figure legend, table legend, main text, or Methods section.

n/a	Confirmed	
☐	☒	The exact sample size (n) for each experimental group/condition, given as a discrete number and unit of measurement
☐	☒	A statement on whether measurements were taken from distinct samples or whether the same sample was measured repeatedly
☐	☒	The statistical test(s) used AND whether they are one- or two-sided<br>*Only common tests should be described solely by name; describe more complex techniques in the Methods section.*
☐	☒	A description of all covariates tested
☐	☒	A description of any assumptions or corrections, such as tests of normality and adjustment for multiple comparisons
☐	☒	A full description of the statistical parameters including central tendency (e.g. means) or other basic estimates (e.g. regression coefficient) AND variation (e.g. standard deviation) or associated estimates of uncertainty (e.g. confidence intervals)
☐	☒	For null hypothesis testing, the test statistic (e.g. F, t, r) with confidence intervals, effect sizes, degrees of freedom and P value noted<br>*Give P values as exact values whenever suitable.*
☒	☐	For Bayesian analysis, information on the choice of priors and Markov chain Monte Carlo settings
☒	☐	For hierarchical and complex designs, identification of the appropriate level for tests and full reporting of outcomes
☐	☒	Estimates of effect sizes (e.g. Cohen's d, Pearson's r), indicating how they were calculated

Our web collection on statistics for biologists contains articles on many of the points above.

Software and code

Policy information about availability of computer code

Data collection	Experimental control and image syntheses conducted with Matlab 2019b, 2020a, 2020b, 2021a, Python 3.7, and MonkeyLogic release v2.0 (NIHM)<br>Data collected using Plexon Inc. Omniplex Neural Recording Data Acquisition System and software, release 19 and 20<br>Eye movement data collection used via ISCAN hardware and software
Data analysis	Analysis code available at https://github.com/PonceLab/as-simple-as-possible

For manuscripts utilizing custom algorithms or software that are central to the research but not yet described in published literature, software must be made available to editors and reviewers. We strongly encourage code deposition in a community repository (e.g. GitHub). See the Nature Portfolio guidelines for submitting code & software for further information.

Data

Policy information about availability of data

All manuscripts must include a data availability statement. This statement should provide the following information, where applicable:

- Accession codes, unique identifiers, or web links for publicly available datasets
- A description of any restrictions on data availability
- For clinical datasets or third party data, please ensure that the statement adheres to our policy

Data availability All correspondence and material requests should be addressed to the corresponding author (C.R.P). Data generated in this study have been deposited in an Open-Science Framework repository71 at the URL https://osf.io/z6gv2/. We may share more data in the future, so a timestamped version of the

repository at the time of publication is also found at https://osf.io/z6gv2/. Data can be obtained by downloading a zip file and can be loaded using MATLAB. Raw spike data and peristimulus histograms are undergoing analyses and are not yet available. Processed data to illustrate each analysis and figure are available at Github (https://github.com/PonceLab/as-simple-as-possible). This Github repository is linked to the aforementioned Open-Science Framework repository. We have shared the latent vectors obtained during image synthesis experiments and responses to the images evoked by those latent vectors, along with sufficient metadata to reproduce most of the results in the publication. Data provided in the OSF repository can be used to replicate Figure 1e,f and Figure 2a,b. In conjunction with code from the GitHub repository, the data can also be used to recreate Figure 2d,e, and Figure 3c,e. Results featured in Figure 4 are based on eye movements applied to images for which we hold no copyright and include pictures depicting identifiable humans, who have not consented for the distribution of their pictures in the context of this work. Thus we have not released the gaze behavior data, nor the baseline reference images due to concerns about privacy and distribution of possibly copyrighted material. However, all of these data are available upon request for research purposes, with guarantees of confidentiality, by contacting the corresponding author. Source data for (supplementary) tables are very large and will be available upon request by contacting the corresponding author. Data used for stimuli and analyses include ImageNet (https://image-net.org/) and COCO-Stuff (https://github.com/nightrome/cocostuff). Source data are provided with this paper.

Field-specific reporting

Please select the one below that is the best fit for your research. If you are not sure, read the appropriate sections before making your selection.

☒ Life sciences ☐ Behavioural & social sciences ☐ Ecological, evolutionary & environmental sciences

For a reference copy of the document with all sections, see nature.com/documents/nr-reporting-summary-flat.pdf

Life sciences study design

All studies must disclose on these points even when the disclosure is negative.

Sample size	In NHP electrophysiology studies, it is standard practice to collect information from tens to hundreds of cells from a single animal. However, individual animals vary in anatomy, genetics and development. Scientific standards require evidence that results observed in a given neuronal population are not exclusive to the individual organism, as could occur through a quirk of developmental life history. Thus a minimum of two to three monkeys are required for any conclusion to be credible and publishable (two monkeys if the experimental findings are concordant; a third is required in case they are not). Illustrations of this approach include Hubel DH, Wiesel TN. Receptive fields and functional architecture of monkey striate cortex. J Physiol. 1968 Mar;195(1):215-43. doi: 10.1113/jphysiol.1968.sp008455. PMID: 4966457; PMCID: PMC1557912.; Desimone R, Albright TD, Gross CG, Bruce C. Stimulus-selective properties of inferior temporal neurons in the macaque. J Neurosci. 1984 Aug;4(8):2051-62. doi: 10.1523/JNEUROSCI.04-08-02051.1984. PMID: 6470767; PMCID: PMC6564959.; Britten KH, Newsome WT, Shadlen MN, Celebrini S, Movshon JA. A relationship between behavioral choice and the visual responses of neurons in macaque MT. Vis Neurosci. 1996 Jan-Feb;13(1):87-100. doi: 10.1017/s095252380000715x. PMID: 8730992.
Data exclusions	After implantation, some array channels were not reliably visually responsive, due to the nature of the array as "floating" (able to move orthogonally to the cortical band), which allows electrode tips to rest outside of the cortical band, or to other factors. We excluded attempted image-synthesis experiments from these channels (corresponding to 12 experiments out of 137 in monkey B), from all analyses except those in the compressibility ratio studies and the gaze behavior studies, as these 12 experiments served in producing 'shuffled prototype' controls.
Replication	Each experiment attempted image-synthesis using single channels in chronically implanted multielectrode arrays (N=64 per animal). Across all experiments, image synthesis successes were achieved / replicated 96.6% [monkey A] and 91.4% [monkey B] in V1/V2, 85.7% and 93.3% in V4, and 71.4% and 83.3% in IT.
Randomization	We used two animals, and they were allocated to the same group. By using chronically implanted arrays without prior functional imaging guidance, we achieved a random sampling of neuronal responses in cortex. All sites were tested using the same experimental protocol. This experimental protocol design used pseudo-random block presentation of images for image selectivity. The image synthesis experiments were also based on stochastic algorithms, and depended on each neuronal site responses. The use of two animals iallows for covariate control, because the inability to replicate results in one animal's neuronal population using a second animal's neuronal population would suggest that the effects are specific to the particular animal.
Blinding	Blinding was not necessary because investigators cannot control the outcome of each experimental session, as the results are based on a closed feedback loop between neurons and adaptive image generators. In experimental projects like this, there is no intervention group or control group, no test treatment or placebo. By testing the entire randomly sampled population with the same experimental design, dependent variables are reliable.

Reporting for specific materials, systems and methods

We require information from authors about some types of materials, experimental systems and methods used in many studies. Here, indicate whether each material, system or method listed is relevant to your study. If you are not sure if a list item applies to your research, read the appropriate section before selecting a response.

Materials & experimental systems

n/a	Involved in the study
☒	☐ Antibodies
☒	☐ Eukaryotic cell lines
☒	☐ Palaeontology and archaeology
☐	☒ Animals and other organisms
☒	☐ Human research participants
☒	☐ Clinical data
☒	☐ Dual use research of concern

Methods

n/a	Involved in the study
☒	☐ ChIP-seq
☒	☐ Flow cytometry
☒	☐ MRI-based neuroimaging

Animals and other organisms

Policy information about studies involving animals; ARRIVE guidelines recommended for reporting animal research

Laboratory animals	Two male 8-year-old rhesus macaques were used
Wild animals	Nonr
Field-collected samples	None
Ethics oversight	All procedures were approved by the Washington University School of Medicine Institutional Animal Care and Use Committee

Note that full information on the approval of the study protocol must also be provided in the manuscript.
